# Preparation and Performance Characterization of Melamine Resin-Coated Water-Based Primer Microcapsule–Brass Powder–Water-Based Acrylic Coating

**DOI:** 10.3390/polym18141773

**Published:** 2026-07-20

**Authors:** Xue Chen, Yan Han, Xiaoxing Yan

**Affiliations:** 1 Co-Innovation Center of Efficient Processing and Utilization of Forest Resources, Nanjing Forestry University, Nanjing 210037, China; chenxue830@njfu.edu.cn (X.C.); hanyan@njfu.edu.cn (Y.H.); 2 College of Furnishings and Industrial Design, Nanjing Forestry University, Nanjing 210037, China

**Keywords:** self-repairing microcapsules, melamine resin, water-based primer, brass powder

## Abstract

The method of how microcapsules and brass powder are added significantly affects the overall effectiveness of decorative coatings. An innovative self-repairing decorative coating for Basswood surfaces was created using melamine resin-coated water-based primer microcapsules as the repair agent under the coating process of “three coats of primer, two coats of topcoat, and introducing brass powder and microcapsules into the primer”. The optical and mechanical properties of this coating were significantly impacted by the curing temperature and brass powder content, respectively. The optical qualities, mechanical properties, liquid resistance, aging resistance, and self-repairing performance of the coating improved at higher curing temperatures under varying brass powder contents. The water-based acrylic wood coating obtained at 60 °C with 3% brass powder and 3% primer microcapsules, featuring a core-wall ratio of 0.58:1, provided the best overall qualities. It has a gloss of 34.1 GU, color difference of 0.00, visible light reflectance of 0.6038, visible light transmittance of 76.20%, color main wavelength of 589.79 nm, hardness of HB, impact resistance of 2 kg·cm, adhesion of grade 1, roughness of 1.125 µm, excellent aging and liquid resistance, and repair rate of 22.93%. This study provides an effective and new strategy for simultaneously improving the decorative, mechanical, and self-repairing properties of water-based wood coatings.

## 1. Introduction

Compared with ordinary colored coatings, metal powder decorative coatings demonstrate significant performance advantages. Their color retention is more durable. Even when exposed to direct sunlight or a humid environment for extended periods, they effectively resist fading. They also exhibit outstanding high-temperature resistance, with the ability to withstand elevated temperatures without cracking or discoloration. In addition, they offer superior durability, significantly extending the service life of the coating [1,2,3]. When applied to the exterior walls of wooden buildings and the surfaces of indoor furniture [4,5,6], these coatings impart a brilliant metallic luster to the otherwise simple wood texture, significantly enhancing the quality of wooden products [7,8]. Therefore, they are highly favored by consumers and have become a premium coating material in the decoration industry [9]. Beyond their decorative appeal, metal powder decorative coatings also show considerable potential in various functional fields, including both civil and military applications, indicating broad prospects for future development [10].

Water-based coatings, with a series of outstanding properties, have already become an important direction for the development of the current coatings industry [11,12,13]. They not only exhibit high hardness but also form a robust protective film after curing, effectively resisting daily friction and impact. In addition, they provide excellent gloss, capable of presenting either a full, transparent mirror-like effect or a soft, delicate matte texture. More importantly, water-based coatings use water as the dispersion medium and therefore emit extremely low levels of volatile organic compounds (VOCs). This significantly reduces environmental pollution throughout the entire process from production to application and also lowers health risks for both workers and end users, perfectly aligning with the current concept of green and environmentally friendly development. In addition to their environmental advantages, water-based coatings offer relatively low production costs and flexible application methods. Under appropriate curing conditions, rapid film formation can be achieved, improving coating efficiency and shortening the construction period [14,15].

Among metallic pigments, brass powder is considered a promising decorative component for water-based wood coatings due to its distinctive golden hue and compatibility with wood substrates. Compared with aluminum powder, which generally exhibits a cool silver-white tone, and other copper-based pigments that may suffer from reduced color stability under long-term environmental exposure, brass powder provides a relatively stable warm golden hue that harmonizes well with the natural color and texture of wood. Moreover, the excellent metallic luster and light-reflective characteristics of brass powder enable the coating to achieve a luxurious decorative effect while preserving the visual depth of wood grain. Although metalized mica pigments possess advantages such as low density and regular platelet morphology, their optical appearance mainly originates from light interference and multiple reflections within the coated mica structure, which generates pearlescent and soft metallic effects rather than the direct metallic luster produced by metal powders [16]. Therefore, brass powder is chosen in this work to achieve an authentic metallic texture, enhanced decorative performance, and practical applicability in water-based wood coatings. In addition, brass powder benefits from relatively low cost and good commercial availability, further supporting its potential for large-scale application.

Microcapsule technology is a technique in which core materials that require protection or controlled release are encapsulated within a high-molecular-weight polymer wall with an isolating function through a specific process, thereby forming microcapsules with diameters typically ranging from a few micrometers to several hundred micrometers. Microcapsules with special functions, such as antibacterial, flame-retardant, color-changing, and self-repairing, are still widely studied and applied today [17,18,19]. Wood is a porous and heterogeneous material that is highly susceptible to environmental influences. The coating applied to the wood surface is particularly susceptible to the inherent shrinkage and swelling behavior of wood under dry and wet conditions. This can lead to the formation of micro-cracks and progressive deterioration, ultimately compromising both its decorative and protective functions. Therefore, self-repairing microcapsule technology can be utilized by incorporating self-repairing microcapsules into water-based wood coatings for application on wood surfaces. This approach enhances the coating’s ability to inhibit and repair micro-cracks, thereby improving the overall protection of both the coating and the wood substrate. Thakur et al. [20] fabricated an epoxy resin coating containing self-repairing microcapsules on the surface of low-carbon steel. The microcapsules were prepared using melamine resin as the shell material, with rosin-based epoxy resin and a rosin imine curing agent as the core materials. Artificial scratch tests showed that the scratches could be repaired within 24 h. Chang et al. [21] developed a self-repairing decorative coating system by encapsulating water-based topcoat within melamine–formaldehyde microcapsules and incorporating KH570-modified brass powder into a water-based acrylic topcoat matrix. The resulting coating exhibited multifunctional performance; however, its optical properties and self-repairing efficiency remained limited. The study was conducted under room-temperature curing conditions, and the influence of curing temperature on the crosslinking behavior of the acrylic resin and the associated structure–property relationships was not systematically investigated. In addition, previous studies [22] indicated that incorporating microcapsules into the primer layer may improve both optical appearance and self-repairing performance compared with incorporation into the topcoat. However, this strategy had not been systematically combined with a brass powder decorative system nor evaluated under controlled curing conditions.

To address these limitations, this work developed a primer-based microcapsule system in which water-based acrylic primer served as the core material. The resulting microcapsules, together with KH570-modified brass powder, were incorporated into a water-based acrylic primer coating to construct a dual-functional decorative and self-repairing system. A “three primer layers and two topcoat layers” architecture was employed to fabricate brass powder-modified wood coatings on basswood substrates. A systematic orthogonal experimental design was first conducted to evaluate the effects of brass powder content, microcapsule core–wall ratio, microcapsule content, and curing temperature on coating performance. Considering the critical role of curing conditions in determining the crosslinking behavior of acrylic resins, curing temperature was selected as a representative parameter to systematically investigate structure–property relationships. The effects on optical, mechanical, durability, and self-repairing properties were evaluated via a controlled single-factor study. Overall, this work introduced a system-level integration of microcapsule chemistry, decorative functionality, and a curing-temperature-regulated structure–property framework, enabling simultaneous optimization of multifunctional performance in wood coatings. Accordingly, it provided a viable strategy for designing multifunctional water-based coatings for wood-based substrates.

## 2. Materials and Methods

### 2.1. Test Materials

The detailed list of test materials is shown in Table 1.

### 2.2. Modification Methods of Brass Powder

According to a mass ratio of ethanol to water of 4:1, 80.0 g of anhydrous ethanol and 20.0 g of deionized water were mixed in a beaker. The mixture was stirred thoroughly with a glass rod and used as the solvent for the hydrolysis reaction of silane. Monohydrate citric acid was then added to adjust the pH of the solution to approximately 5. Subsequently, 0.6 g of KH570 modifier was slowly added dropwise under continuous stirring to obtain the silane-modified solution. After that, 10.0 g of brass powder was added to the silane-modified solution. The mixture was stirred at 600 r·min^−1^ in a 30 °C water bath for 3 h. After the reaction, the unreacted silane-modifier was removed by repeated washing with deionized water and anhydrous ethanol, followed by filtration. Finally, the product was dried in a 60 °C oven to obtain the modified brass powder.

### 2.3. Preparation of Microcapsules of Water-Based Primer Coated with Melamine Resin

(1) Preparation of a Mixture of Hydroxymethyl Melamine

According to Table 2, melamine and 37% formaldehyde solution were weighed, and deionized water was added. Triethanolamine was then added dropwise to adjust the pH of the mixture to 8–9. Subsequently, the mixture was stirred at 70 °C and 600 rpm for 30 min to obtain a water-soluble hydroxymethyl melamine solution.

(2) Emulsification of Core Material

Firstly, 89.7 g of deionized water was weighed and added to a beaker. Then, 0.3 g of SDBS was introduced, and the mixture was stirred evenly with a glass rod to obtain an emulsifier solution. A total of 10.0 g of water-based primer was added dropwise into the emulsifier solution. The beaker was placed in a constant-temperature water bath, and the mixture was stirred at 70 °C and 600 rpm for 60 min to obtain a uniformly dispersed core material emulsion.

(3) Molding, Coating, and Drying of Microcapsules

The prepolymer solution was slowly added dropwise into the core material emulsion at 600 rpm. The mixture was then subjected to ultrasonic dispersion using an ultrasonic emulsifying and dispersing device for 15 min. Subsequently, a 20 wt% citric acid monohydrate solution was added dropwise to adjust the pH to approximately 3. The reaction system was heated in a water bath and maintained at 60 °C for 3 h under constant stirring. Afterward, the mixture was left to stand at 20 °C for 3 days. The resulting product was then repeatedly washed with deionized water and anhydrous ethanol, followed by vacuum filtration. Finally, the collected microcapsules were dried in an oven at 60 °C for 24 h to obtain dried microcapsules in powder form.

### 2.4. Preparation of Self-Repairing Brass Powder–Water-Based Acrylic Coating

**Figure 1:**
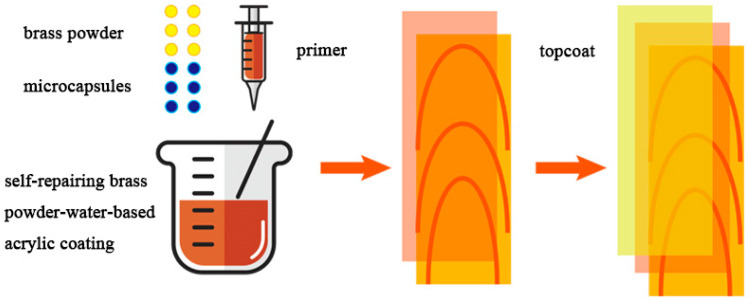
Flowchart for the preparation of self-repairing brass powder–water-based acrylic coating on the wood surface.

Firstly, the modified brass powder and the prepared water-based primer microcapsules were added to a water-based acrylic primer coating at a certain mass content, and the mixture was stirred thoroughly with a glass rod to obtain 2.0 g of primer coating. The primer coating was then evenly divided into three portions and sequentially applied in layers onto the surface of basswood substrates. After each coating application, the samples were cured and dried in an oven at different curing temperatures according to the experimental design. Each dried layer was subsequently polished with 1000-mesh sandpaper to ensure a smooth surface before the next application. After the third primer layer was completed, 2.0 g of water-based topcoat was prepared and uniformly applied in two coats on top of the primer system. The coated samples were then placed in an oven and cured at a specified temperature until the mass remained constant, after which they were removed. Figure 1 shows a flowchart for the preparation of self-repairing brass powder–water-based acrylic coating on the wood surface.

### 2.5. Orthogonal Experimental Design

To systematically investigate the effects of brass powder content, microcapsule parameters, and curing conditions on the comprehensive performance of the coating, a four-factor, three-level L9(3^4^) orthogonal experimental design was adopted. The factor levels were determined based on preliminary experimental results and relevant literature.

Previous studies [23] had shown that KH570-modified brass powder achieved optimal overall performance at a loading of approximately 10%, exhibiting good film-forming ability and dispersion stability; however, further increases in filler content could compromise coating continuity and mechanical properties. Therefore, the brass powder content was limited to below 10%, and three gradient levels of 3%, 6%, and 9% were selected to represent low, medium, and high filler contents. For self-repairing coatings, both the core–wall ratio of microcapsules and their content [24] significantly affected coating performance and repairing efficiency. Core–wall ratios in the range of 0.58:1–0.75:1 were frequently used in microcapsule preparation to balance encapsulation efficiency and structural stability. Previous work [25] indicated that microcapsule loading was limited by the overall coating performance; to balance mechanical properties and self-repairing functionality, the content was also restricted to below 10%, and three levels of 3%, 6%, and 9% were selected for investigation. In addition, curing temperature was a critical processing parameter influencing the performance of wood coatings [26]. Considering the thermal sensitivity and dimensional stability of wood substrates, the curing temperature was generally kept below 60 °C. Accordingly, room temperature (approximately 25 °C), 40 °C, and 60 °C were selected as the three levels.

In summary, four factors were selected: KH570-modified brass powder content (3%, 6%, 9%), microcapsule core–wall ratio (0.58:1, 0.67:1, 0.75:1), microcapsule content (3%, 6%, 9%), and curing temperature (25 °C, 40 °C, 60 °C), and a L9(3^4^) orthogonal experimental design was constructed (Tables S1 and S2). The detailed dosage of the coating system in the orthogonal experiment is shown in Table S3.

### 2.6. Design of Single-Factor Experiment

The prepared self-repairing brass powder–water-based acrylic coating samples were subjected to orthogonal analysis using two evaluation indicators, namely gloss and elongation at break, to identify the most significant influencing factor. Based on the results, curing temperature was selected as the main factor for further investigation (30, 35, 40, 45, 50, 55, and 60 °C), and a single-factor experiment was subsequently conducted. Under the condition of adding 3% microcapsules with a core-wall ratio of 0.58:1, the effect of curing temperature on the performance of self-repairing water-based acrylic coatings containing different contents of brass powder was evaluated (Table 3). The coating procedure for all samples was consistent, consisting of three primer layers and two topcoat layers, with both primer microcapsules and modified brass powder incorporated into the primer simultaneously.

## 3. Testing and Characterization

### 3.1. Encapsulation Efficiency Test of Microcapsules

Encapsulation efficiency was determined by weighing a microcapsule sample with a mass of *P*_1_. The sample was thoroughly ground and soaked in ethanol, and then allowed to stand for 48 h. After that, it was washed and filtered using a suction filtration system. The filter paper and the solid product obtained after filtration were transferred to a 60 °C oven and dried to constant mass, which was recorded as *P*_2_. The encapsulation efficiency was calculated according to Formula (1).

Encapsulation Efficiency = (*P*_1_ − *P*_2_)/*P*_1_ × 100% (1)

### 3.2. Characterization of Macroscopic and Microscopic Morphologies and Chemical Composition

The macroscopic morphology of the coated Basswood surface after finishing was recorded using a SONY Alpha 7C camera (Sony Co., Ltd., Beijing, China). The morphology of microcapsules was observed using an Axio Lab. A1 optical microscope (OM, Beijing Presys Instrument Co., Ltd., Beijing, China), and the microscopic morphologies of both the microcapsules and coatings were characterized by scanning electron microscopy (SEM). The particle size distribution of microcapsules was analyzed using Nano Measurer software (v1.2) [27], based on a sample size of 100 particles. The chemical compositions of microcapsules and coating samples were characterized using Fourier transform infrared spectroscopy. Fourier transform infrared (FTIR) spectra were recorded using a VERTEX 80V spectrometer (Bruker, Germany). For microcapsule samples, the spectra were collected using the KBr pellet method (transmission FTIR mode) in the range of 4000–400 cm^−1^. The coating samples were analyzed using an attenuated total reflectance (ATR) accessory within the same wavenumber range.

### 3.3. Testing of Optical Properties

According to GB/T 4893.6-2013 [28], the gloss of the coatings was measured using a touch-screen three-angle gloss meter (NHG268, Shenzhen Threenh Technology Co., Ltd., Shenzhen, China). The gloss values were recorded at incident angles of 20°, 60°, and 85°, respectively, and reported in gloss units (GU).

The color difference (Δ*E*) of the coating was measured using a SEGT-J type 3nh three-angle color difference meter (Shenzhen Threenh Technology Co., Ltd., Shenzhen, China). To ensure reliability, at least three measurements were performed at randomly selected positions on each coating surface, and the chromaticity parameters (L*, a*, b*) were recorded. The average values of L*, a*, and b* were used for subsequent analysis. The color difference (Δ*E*) of the coating was then calculated according to Formula (2) [29,30].

(2) $$\Delta E={\left[{\left(\Delta L\right)}^{2}+{\left(\Delta a\right)}^{2}+{\left(\Delta b\right)}^{2}\right]}^{1/2}$$

In addition, a color difference meter was used to measure the chromaticity values of water-based coatings containing different contents of brass powder. Four randomly selected points were tested for each coating, and the average values were calculated as the chromaticity parameters (L*, a*, and b*), respectively. The L*, a*, and b* values were then subtracted from those of the control coating without brass powder to obtain ΔL*, Δa*, and Δb*. The chromaticity changes of coatings with different contents of brass powder were calculated according to the color difference Formula (2), denoted as ΔE* [31,32]. Furthermore, ΔE* was determined based on the variation between the initial and final states of the coating samples under different conditions, including curing temperature and aging treatment.

The wavelength-reflectance curves, dominant wavelength data, and color saturation of the coatings on the Basswood surface within the visible light wavelength range (380–780 nm) were obtained using a Hitachi U-3900/3900H UV–visible spectrophotometer (Hitachi Instruments Co., Ltd., Suzhou, China). According to ASTM G173-03 [33], the solar reflectance (R) of the coatings within the visible light range was calculated by Formula (3), where i(λ) represents the standard solar spectral irradiance (W·m^−2^·nm^−1^), and r(λ) represents the measured reflectance.

(3) $$R=\frac{\int_{380}^{780}r\left(\lambda \right)i\left(\lambda \right)d\left(\lambda \right)}{\int_{380}^{780}i\left(\lambda \right)d\left(\lambda \right)}$$

In addition, the wavelength-transmittance curves of the coatings on the Basswood surface within the visible light wavelength range (380–780 nm) were obtained using a Hitachi U-3900/3900H UV–visible spectrophotometer. The solar transmittance (τ) of the coatings within the visible light range was calculated by Formula (4) to characterize coating transparency. Here, d(λ)v(λ) is the weighting factor derived from the spectral distribution of CIE daylight D_65_ and CIE photopic standard relative luminosity; τ_t_ is the spectral transmittance at each wavelength, expressed as a percentage (%).

(4) $$\tau =\frac{\sum_{\lambda }d\left(\lambda \right)v(\lambda )\tau_{t}(\lambda )}{\sum_{\lambda }d\left(\lambda \right)v(\lambda )}$$

### 3.4. Testing of Coating Hardness

According to GB/T 6739-2006 [34], the coating hardness was evaluated using pencil hardness testing with pencils ranging from 6B to 6H, performed on a QHQ-A portable pencil hardness tester (Liangchuang Instrument Co., Ltd., Suzhou, China). The pencils were inserted obliquely at a 45° angle into the pencil-type hardness tester with a load of 750 g. The hardness of the pencils was gradually increased, starting from 6B until permanent indentations appeared on the coating samples, and the pencil hardness at this point was taken as the coating hardness.

### 3.5. Testing of Impact Resistance of Coatings

According to the standard GB/T 4893.9-2013 [35], the impact resistance of the coatings was evaluated using a BEVS 1601 impact tester (BEVS Industrial Co., Ltd., Guangzhou, China). The finished Basswood boards were placed on the tester, and a steel ball was positioned directly above the coated surface. The ball was then raised to a predetermined height and released to freely fall and impact the boards. The minimum height at which coating damage occurred was recorded as the impact resistance value of the coating, expressed in kg·cm.

### 3.6. Testing of Coating Adhesion

According to the standard GB/T 4893.4-2013 [36], the adhesion of the coatings was evaluated using a QFH-HG600 cross-cut tester (Dongguan Zhongte Automation Technology Co., Ltd., Dongguan, China). The blade of the cross-cut tester was held perpendicular to the coating surface, and a uniform force was applied manually to produce a set of parallel cutting lines on the coating surface. The cutting procedure was then repeated at a 90° angle to the first set of lines, forming a grid pattern. An adhesive tape (approximately 100 mm in length) was applied to cover the entire grid, and after 5 min, the tape was smoothly peeled off by hand. The adhesion grade was determined based on the amount of coating detached onto the tape. The grades were classified from 1 to 5, where grade 1 indicates the best adhesion and higher values represent progressively poorer adhesion.

### 3.7. Testing of Coating Roughness

A J8-4C surface roughness tester (Shanghai Taiming Optical Instrument Co., Ltd., Shanghai, China) was used to measure the surface roughness. The finished Basswood board was placed on the testing platform, and the probe was brought into contact with the surface. After adjusting the probe position and stabilizing it at the zero coordinate, the measurement was initiated. The surface roughness (Ra) was then recorded, with a unit of μm.

### 3.8. Testing of Aging Resistance of Coatings

The coating samples were placed in an oven at 160 °C for high-temperature accelerated aging to evaluate their dry heat aging resistance. In addition, according to ASTM D4587-2011 [37], UVA-340 fluorescent lamps were used to conduct an ultraviolet (UV) photooxidation resistance test on the prepared coating samples, with three cycles of the operation steps of “4 h of irradiation (0.89 W·m^−2^·nm^−1^) and 20 h of condensation”. The chromaticity change value ΔE* of the coatings was calculated using Formula (2) to evaluate their aging resistance.

### 3.9. Testing of Coating Resistance to Cold Liquids

According to the standard GB/T 4893.1-2021 [38], a 10% mass fraction citric acid aqueous solution, 96% volume fraction undenatured ethanol, dishwashing detergent, and coffee (prepared by dissolving 40 g of freeze-dried instant coffee in 1 L of boiling water) were selected as test solutions. Filter papers (25 mm in diameter) were soaked in the corresponding solutions, removed, and placed on the coating surface. The samples were then stored in a closed environment. After 24 h, the filter papers were removed, and the samples were left to stand for an additional 16 h. The surface damage was then visually assessed, and the resistance grade was determined according to the grading table (Table 4). A color difference meter was used to record the chromaticity values of the coatings on the Basswood surface before and after the liquid resistance test, and the chromaticity change value ΔE* was calculated according to Formula (2).

### 3.10. Testing of Self-Repairing Performance of Coatings

The change rate of scratch width is commonly used to evaluate the self-healing performance of coatings. However, it may also be influenced by various factors, including temperature, humidity, coating thickness, and chemical composition. To ensure the reliability of this evaluation method, a ZBQ four-sided film applicator (Pushen Testing Instruments Co., Ltd., Shanghai, China) was used to control the coating thickness at 60 μm and maintain a consistent formulation of the coatings. In addition, all tests were conducted in an LT-TH-80 constant temperature and humidity chamber (Dongguan Lituo Testing Instruments Co., Ltd., Dongguan, China).

During the scratch test, the wood exhibited no obvious shrinkage or swelling due to moisture variation, and the coating remained in good condition. A Feiying 74-C single-sided safety blade (Shanghai Gillette Co., Ltd., Shanghai, China) was used to create a scratch with a depth of 60 µm on the self-repairing coating, ensuring that the coating sample was just scratched. An optical microscope (OM) was used to observe the changes in the coating scratch immediately after it was created and after being left to stand in a constant temperature and humidity chamber (temperature: 25 °C, humidity: 45%) for 3 days. The scratch width change rate (*D_H_*) was calculated according to Formula (5) to characterize the self-repairing performance of the coating. Here, *D_H_* represents the width change rate in %; *D*_1_ is the initial width of the scratch on the Basswood surface coating in µm; and *D*_2_ is the width of the scratch on the Basswood surface coating after 3 days in µm. All measurements were repeated four times, and the experimental error was controlled within 5%.

(5) $$D_{H}=\frac{D_{1}-D_{2}}{D_{1}}\times 100\%$$

## 4. Results and Discussion

### 4.1. Analysis of Microscopic Morphology and Infrared Spectroscopy of Melamine Resin–Water-Based Primer Microcapsules with Different Core-Wall Ratios

(1) Microscopic Morphology

**Figure 2:**
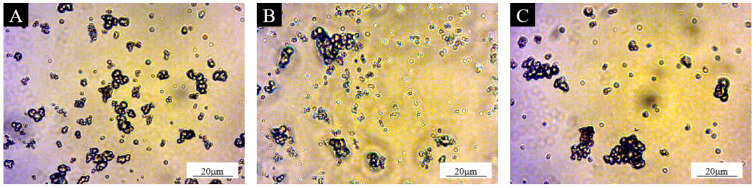
OM diagram of microcapsules with different core-wall ratios: (**A**) 0.58:1, (**B**) 0.67:1, (**C**) 0.75:1.

**Figure 3:**
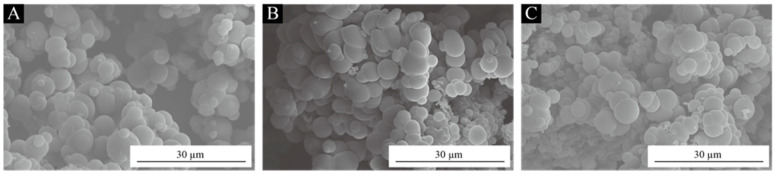
SEM diagram of microcapsules with different core-wall ratios: (**A**) 0.58:1, (**B**) 0.67:1, (**C**) 0.75:1.

**Figure 4:**
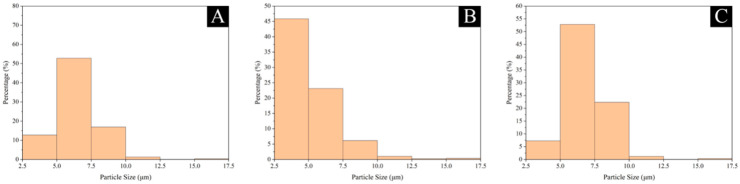
Particle size of microcapsules with different core-wall ratios: (**A**) 0.58:1, (**B**) 0.67:1, (**C**) 0.75:1.

The microscopic morphologies of microcapsules with three different core-wall material ratios are shown in Figure 2 and Figure 3. In Figure 2, ring-like boundaries are observed, indicating that the microcapsules are composed of two different media, with the bright inner part representing the core material (water-based primer) of the microcapsules. Based on the combined SEM and OM results, the self-healing microcapsules with core–wall ratios of 0.58:1, 0.67:1, and 0.75:1 all exhibited a generally spherical morphology. Among them, the microcapsules with ratios of 0.58:1 and 0.75:1 showed relatively well-defined spherical structures with smooth surfaces and intact shapes. In contrast, the microcapsules with a ratio of 0.67:1 displayed less uniform spherical morphology, and noticeable agglomeration was observed. The particle size distributions of microcapsules with three different core-wall material ratios are shown in Figure 4. The microcapsules with core-wall material ratios of 0.58:1 and 0.75:1 exhibited larger and more uniform particle sizes, mainly distributed in the range of 5.0–7.5 μm. By comparison, the microcapsules with a ratio of 0.67:1 showed smaller particle sizes, predominantly distributed in the range of 2.5–5.0 μm.

(2) Analysis of Infrared Spectroscopy

**Figure 5:**
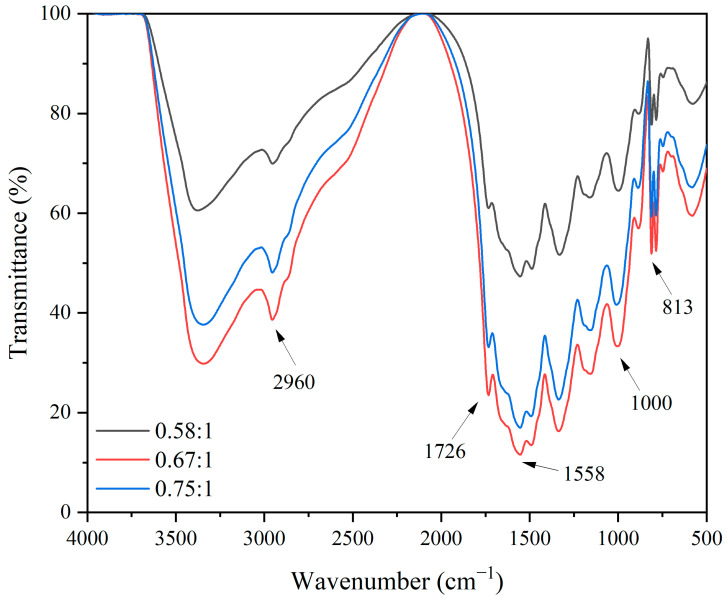
FT-IR spectra of three primer microcapsules with different core-wall ratios.

Figure 5 shows the infrared spectra of three primer microcapsule samples with different core-wall ratios. The FTIR spectra exhibited characteristic absorption bands from both the melamine-formaldehyde shell and the water-based acrylic primer core, confirming the successful formation of core–shell structured microcapsules. Specifically, the broad band at 3300–3450 cm^−1^ was attributed to O–H and N–H stretching vibrations, indicating hydrogen bonding interactions within the microcapsules. The peak at 2960 cm^−1^ was attributed to the C–H stretching vibrations of –CH_2_ and –CH_3_ groups from the acrylic resin core. The strong absorption peak at 1726 cm^−1^ was assigned to the C=O stretching vibration of ester groups in the acrylic resin, confirming the presence of the core material. The band at 1558 cm^−1^ corresponded to N–H bending vibration and C–N stretching of the triazine ring in the melamine resin shell, while the absorption at around 1000–1150 cm^−1^ was associated with C–O vibrations. The characteristic peak at 813 cm^−1^ was attributed to the triazine ring out-of-plane bending vibration of melamine resin.

Although the three microcapsules exhibited similar characteristic absorption bands, differences in transmittance intensity were observed among samples with different core-wall ratios. These variations were mainly related to changes in the relative proportions of acrylic primer core and melamine-formaldehyde wall components, which affected the contribution of their characteristic functional groups to the FTIR spectra. However, the overall transmittance did not exhibit a simple linear relationship with increasing core content, because the combined absorption behavior of all functional groups in the microcapsule system determined the FTIR response. The nearly unchanged peak positions across the three samples indicated that the observed spectral differences arose from variations in component ratios rather than from the formation of new chemical bonds or structural transformations during encapsulation. Therefore, the FTIR results confirmed that both the acrylic primer core and melamine-formaldehyde shell retained their original chemical structures after encapsulation.

(3) Encapsulation efficiency analysis of microcapsules

The encapsulation efficiencies of melamine resin–water-based primer microcapsules prepared with different core-wall ratios are presented in Table 5. As the core-wall ratio increased from 0.58:1 to 0.75:1, the encapsulation efficiency gradually decreased from 54.3% to 36.6%, showing a clear downward trend. The microcapsules prepared at a core-wall ratio of 0.58:1 exhibited the highest encapsulation efficiency, whereas those prepared at 0.75:1 showed the lowest value. This phenomenon is mainly attributed to the variation in the mass ratio between the core and wall materials. As the core-wall ratio increased, a given amount of wall material was required to encapsulate a larger amount of core material, while the relative amount of melamine resin available for wall formation decreased. Consequently, the wall thickness decreased, and part of the water-based primer could not be completely encapsulated, resulting in a lower encapsulation efficiency. In addition, a higher core-wall ratio tended to cause non-uniform deposition of the wall material on the droplet surface during in situ polymerization, leading to wall defects and a further reduction in encapsulation efficiency. Combined with the microscopic morphology results shown in Figure 2 and Figure 3, the microcapsules prepared at a core-wall ratio of 0.58:1 exhibited an intact spherical structure, smooth surfaces, and uniform particle size distribution, whereas those prepared at 0.67:1 showed slight agglomeration and those prepared at 0.75:1 exhibited thinner walls, ultimately resulting in a lower encapsulation efficiency.

### 4.2. Analysis of Orthogonal Test Results

(1) Gloss

**Figure 6:**
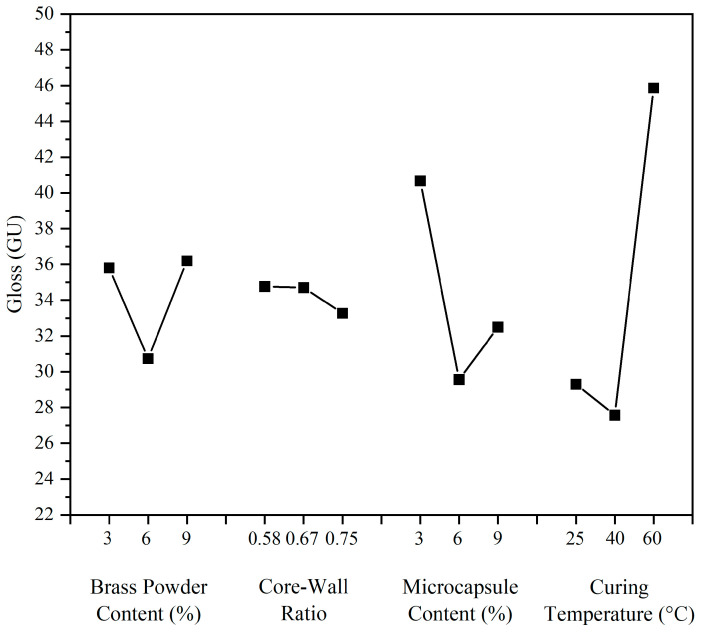
The film’s gloss effect at a 60° incidence angle.

The gloss results of modified brass powder–water-based acrylic decorative coatings containing self-repairing primer microcapsules are presented in Table S4. Sample 8 exhibited the highest gloss (54.7 GU at a 60° incident angle), followed by Sample 3 (44.7 GU). The range analysis results in Table 6 indicated that curing temperature had the greatest influence on coating gloss, followed by microcapsule content, brass powder content, and core–wall ratio. As shown in Figure 6, both curing temperature and microcapsule content exhibited nonlinear effects on gloss. The gloss initially decreased and then increased with increasing curing temperature, with a significant improvement from 40 to 60 °C, which is attributed to enhanced film formation and surface smoothness at higher curing temperatures. The variation in gloss with microcapsule content was mainly related to changes in surface roughness and light scattering caused by dispersed microcapsules. The variance analysis results in Table 7 further confirmed that curing temperature and microcapsule content significantly affected coating gloss. Based on the combined range and variance analyses, the optimal formulation for achieving maximum gloss was determined as 9% brass powder, a core–wall ratio of 0.58:1, 3% microcapsule content, and a curing temperature of 60 °C.

(2) Elongation at Break

**Figure 7:**
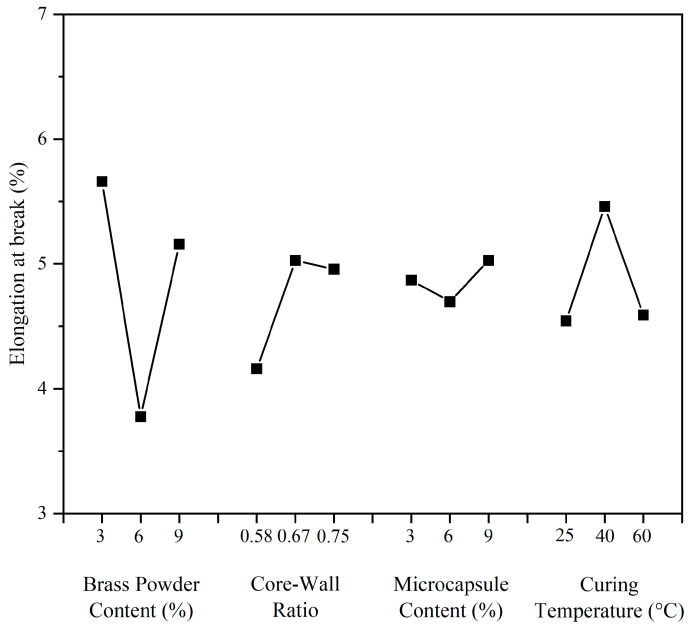
Elongation at break effect curve.

The elongation at break results of the coatings prepared via orthogonal design are presented in Table 8. Sample 2 exhibited the highest elongation at break (6.25%), followed by Sample 7 (5.66%) and Sample 3 (5.64%). Range analysis showed that brass powder content had the greatest influence on elongation at break, followed by curing temperature, core–wall ratio, and microcapsule content. As shown in Figure 7, the elongation at break exhibited a nonlinear variation with brass powder content. It decreased from 5.66% to 3.78% when the brass powder content increased from 3% to 6%, which was mainly attributed to the restricted polymer chain mobility induced by rigid brass powder particles. Previous studies have reported that rigid fillers can limit polymer chain movement and introduce local stress concentration, thereby reducing elongation at break [39]. With a further increase to 9%, the elongation at break slightly recovered, possibly due to changes in particle distribution and stress transfer behaviour within the coating matrix. The core–wall ratio also showed a non-monotonic effect, with 0.67:1 achieving the highest elongation due to the balance between capsule integrity and shell rigidity. A further increase in core–wall ratio may reduce capsule stability due to thinner shells. Meanwhile, curing temperature affected elongation by regulating acrylic resin crosslinking, and an appropriate temperature balanced network formation and chain mobility. Variance analysis (Table 9) further confirmed that brass powder content had the most significant effect on elongation at break. Based on the range and variance analyses, the optimal parameters for elongation performance were determined as 3% brass powder, a core–wall ratio of 0.67:1, 9% microcapsule content, and a curing temperature of 40 °C.

### 4.3. Analysis of Single-Factor Test Results

#### 4.3.1. Analysis of Optical Properties of Coatings

(1) Macroscopic Morphology

**Figure 8:**
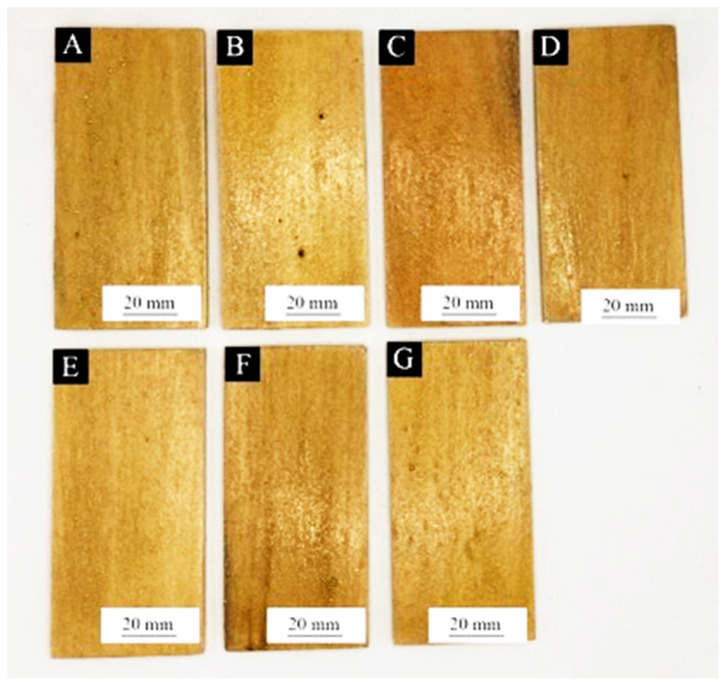
Microcapsule–water-based acrylic coating containing 3% brass powder on basswood surface: (**A**) Sample 10, (**B**) Sample 11, (**C**) Sample 12, (**D**) Sample 13, (**E**) Sample 14, (**F**) Sample 15, and (**G**) Sample 16.

**Figure 9:**
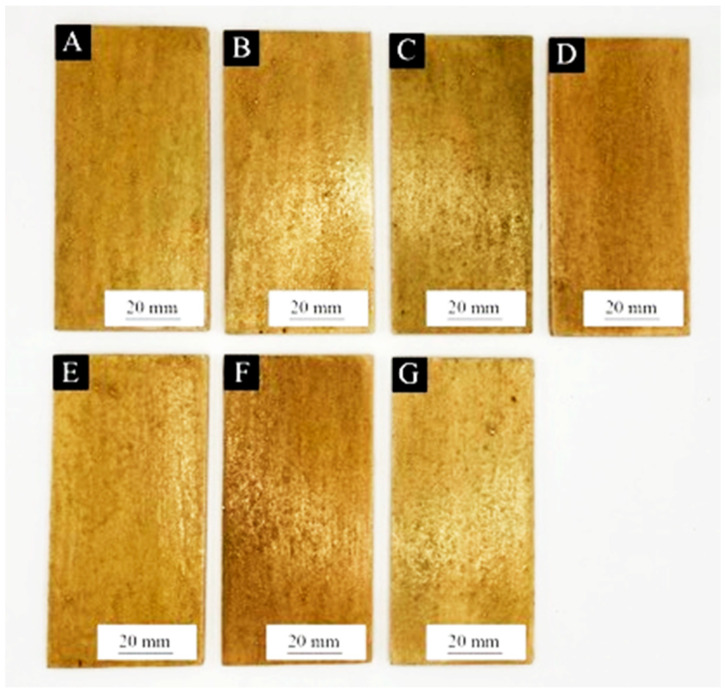
Microcapsule–water-based acrylic coating containing 6% brass powder on basswood surface: (**A**) Sample 17, (**B**) Sample 18, (**C**) Sample 19, (**D**) Sample 20, (**E**) Sample 21, (**F**) Sample 22, and (**G**) Sample 23.

**Figure 10:**
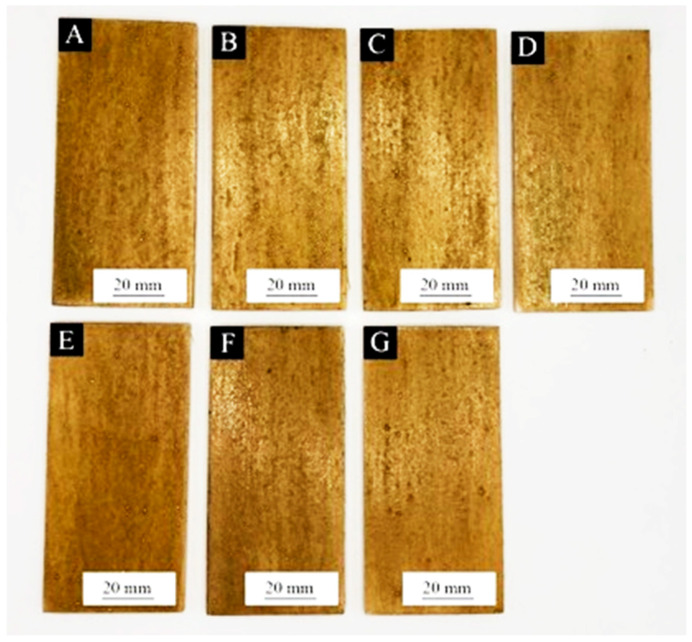
Microcapsule–water-based acrylic coating containing 9% brass powder on basswood surface: (**A**) Sample 24, (**B**) Sample 25, (**C**) Sample 26, (**D**) Sample 27, (**E**) Sample 28, (**F**) Sample 29, and (**G**) Sample 30.

The samples prepared in the single-factor test are shown in Figure 8, Figure 9 and Figure 10. It could be observed from the figures that the microcapsule-water-based acrylic coating containing 3% brass powder exhibited a lighter color, followed by the coating containing 6% brass powder. In contrast, the coating containing 9% brass powder showed the darkest color.

(2) Gloss

**Figure 11:**
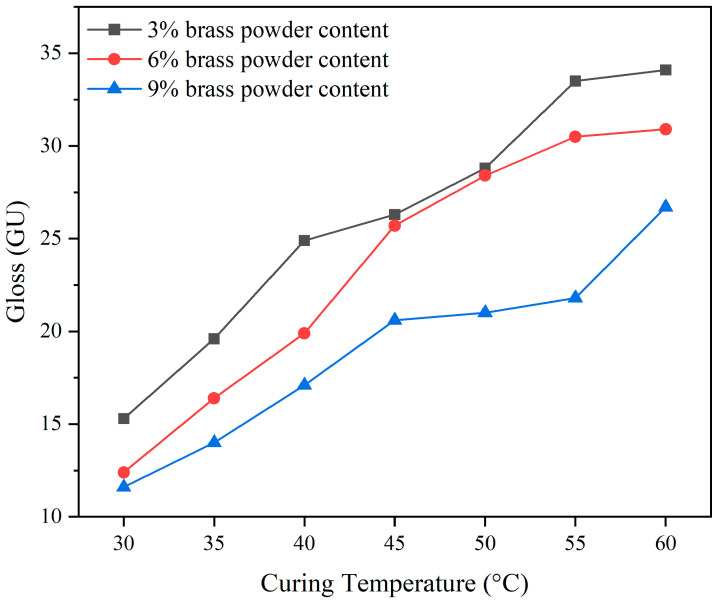
Effect of curing temperature on gloss of decorative coatings with different brass powder contents.

The effect of curing temperature on the gloss of decorative coatings with different contents of brass powder is shown in Figure 11 and Table S5. The self-repairing decorative coatings containing 3% and 6% brass powder both exhibited good gloss performance. When the curing temperature increased from 30 °C to 60 °C, the gloss of the coating containing 3% brass powder increased from 15.3 GU to 34.1 GU, while that of the coating containing 6% brass powder increased from 12.4 GU to 30.9 GU. However, the coating containing 9% brass powder showed relatively lower gloss. Over the same temperature range (30–60 °C), its gloss increased from 11.6 GU to 26.7 GU. Overall, within the temperature range of 30–60 °C, the curing temperature was positively correlated with the gloss of the self-repairing brass powder–water-based acrylic coating. This is because a lower curing temperature would cause residual monomers or other impurities in the water-based acrylic resin during curing, and these unreacted impurities might lead to phenomena such as clouding [40], thereby reducing the gloss. In addition, brass powder content had a noticeable influence on coating gloss, showing a negative correlation. The incorporation of brass powder increased surface roughness, enhancing diffuse reflection when light interacted with the coating surface, which in turn reduced gloss [41].

(3) Color Difference

**Figure 12:**
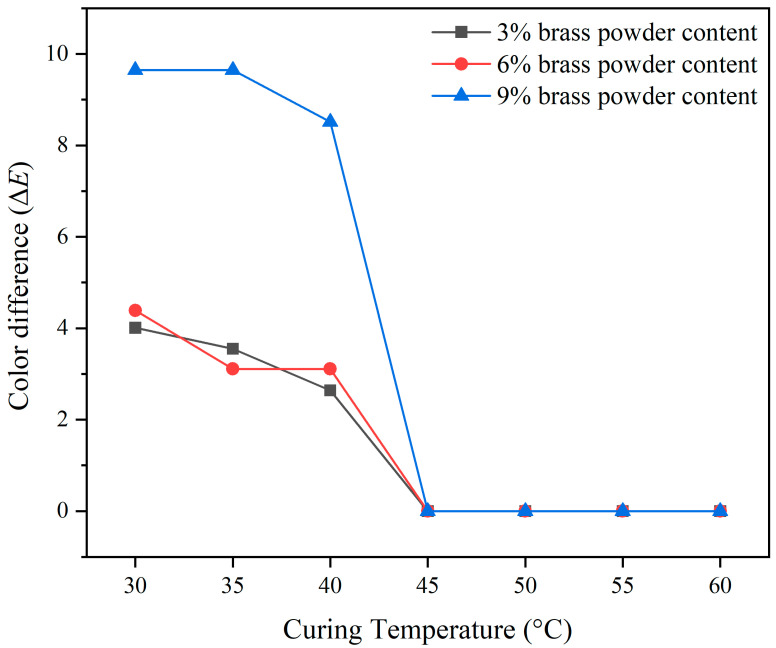
Effect of curing temperature on Δ*E* of decorative coatings with different brass powder contents.

The effect of curing temperature on the color difference of decorative coatings with different contents of brass powder is shown in Table S6 and Figure 12. For self-repairing decorative coatings with varying brass powder contents, curing temperature had a noticeable influence on the color uniformity of the coating surface. In general, lower curing temperatures resulted in larger color differences, whereas higher curing temperatures led to more uniform surface coloration. When the curing temperature exceeded 40 °C, the color differences of all three coatings reached their minimum, which was 0.00. This is because when the water-based acrylic coating for wood was cured at a temperature of 40–60 °C, the resin could be fully crosslinked. However, when the acrylic resin was cured in a low-temperature environment, some monomers and impurities would remain in the cured resin matrix, leading to surface discoloration phenomena such as whitening or yellowing. Consequently, the color uniformity of the coating was reduced.

(4) Visible Light Reflectance

**Figure 13:**
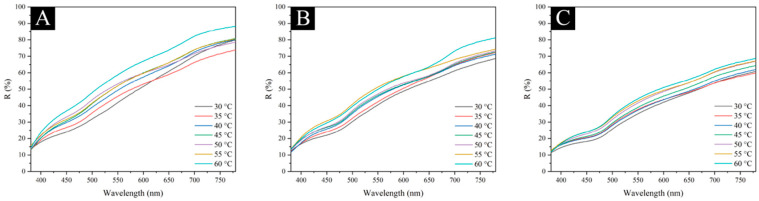
Effect of curing temperature on reflectance of decorative coatings with different brass powder contents: (**A**) 3%, (**B**) 6%, (**C**) 9%.

**Figure 14:**
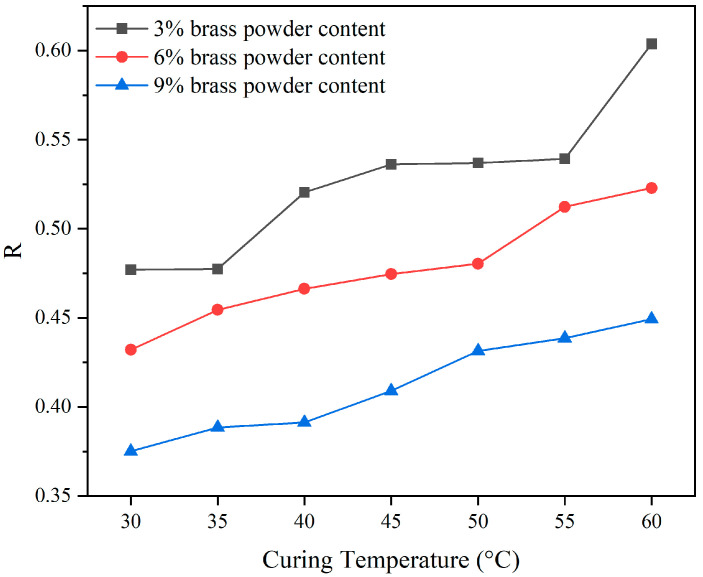
Effect of curing temperature on the R value of coatings with different contents of brass powder.

The effects of different curing temperatures on the reflectance in the visible light band of decorative coatings with different contents of brass powder are reflected in Figure 13, Table S7, and Figure 14. The curing temperature had a significant influence on the reflectance (R value) of brass powder decorative coatings in the visible light band. Meanwhile, the effect of brass powder content on the reflectance of the self-repairing decorative coatings was also pronounced. For self-repairing decorative coatings with a fixed content of brass powder, the curing temperature was positively correlated with the reflectance in the visible light band. In other words, higher curing temperatures resulted in higher reflectance. Among the coatings with different brass powder contents, the sample containing 3% brass powder exhibited the highest reflectance, followed by the 6% sample. The coating with 9% brass powder showed the lowest reflectance. This trend can be attributed to the increasing density of brass powder particles with higher loading. As the content of brass powder increased, the particles became more closely packed within the coating. Consequently, incident light interacted with more brass particles, leading to enhanced light scattering and thus reduced reflectance.

Figure 13 could also reflect the color saturation of the coating samples. The self-repairing decorative coatings with different contents of brass powder showed the same trend in terms of color saturation; that is, as the content of brass powder increased, the coating color became more saturated. The higher the saturation, the more vivid the overall color effect presented by the coating. Among them, the decorative coating containing 3% brass powder and cured at 60 °C showed the highest color vividness.

Table S8 presents the dominant wavelengths of the surface color of self-repairing decorative coatings with different contents of brass powder prepared at different curing temperatures. The dominant wavelengths of all samples ranged from 586.78 nm to 588.07 nm, which fell within the yellow region of visible light (577–597 nm). This indicated that all coatings containing microcapsules with different core–wall ratios exhibited a yellow surface color. With increasing curing temperature, the dominant wavelength shifted closer to 588.07 nm, suggesting that the overall visual color tended toward orange. In contrast, at lower curing temperatures, the dominant wavelength approached 586.78 nm, indicating a shift toward a more yellow appearance.

(5) Visible Light Transmittance

**Figure 15:**
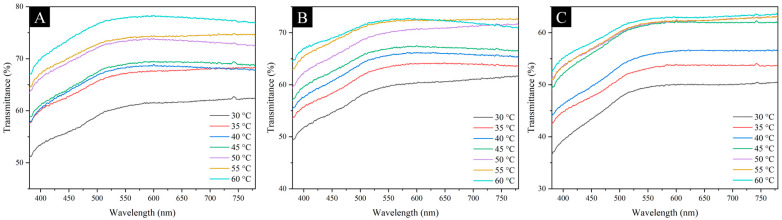
Transmittance curve of decorative coatings with different brass powder contents: (**A**) 3%, (**B**) 6%, (**C**) 9%.

Figure 15 shows the light transmittance curves of self-repairing decorative coatings with different contents of brass powder cured at different temperatures. Table 10 presents the corresponding visible light transmittance values. It could be observed from the figures and table that the light transmittance of all coatings increased continuously with increasing curing temperature. For the coating containing 3% brass powder, the light transmittance increased from 59.69% to 76.20%, with an increase of 16.51%. For the coating containing 6% brass powder, the light transmittance rose from 58.51% to 70.98%, corresponding to an increase of 12.47%. For the coating with 9% brass powder, the light transmittance increased from 47.64% to 61.27%, with an increase of 13.63%. When the curing temperature reached 60 °C, all three coatings exhibited the most pronounced improvement in light transmittance. This may be attributed to incomplete curing at lower temperatures. Inadequate curing of the water-based acrylic resin can lead to residual monomers and impurities, which may cause color deviation and reduce coating transparency.

#### 4.3.2. Analysis of Coating Hardness

The changes in hardness of self-repairing decorative coatings with different contents of brass powder on the surface of the Basswood as a function of curing temperature are shown in Table 11. For the coating containing 3% brass powder, the hardness at 30 °C was the lowest, corresponding to grade B. When the temperature increased to 60 °C, the hardness improved from grade B to grade HB. For coatings with 6% and 9% brass powder, the initial hardness at 30 °C was grade HB. As the curing temperature increased, further improvements were observed. When the temperature rose to 50 °C, the hardness of the 9% brass powder coating increased from HB to H. With the curing temperature increasing to 55 °C, the 6% brass powder coating also improved from HB to H. Overall, for coatings with a given brass powder content, the curing temperature showed a positive correlation with hardness; higher curing temperatures led to higher coating hardness. This is because a lower curing temperature might result in incomplete polymerization of water-based acrylic, leading to reduced resin strength and decreased hardness. In addition, the content of brass powder also influenced coating hardness. Higher brass powder loading generally resulted in higher hardness. This can be attributed to the reinforcing effect of rigid fillers, including both microcapsules and brass powder particles, which enhance the overall mechanical strength of the coating system.

#### 4.3.3. Analysis of Impact Resistance of Coatings

The changes in impact resistance of self-repairing decorative coatings with different brass powder contents on the surface of the Basswood as a function of curing temperature are shown in Table 12. The impact strength of the coatings was influenced to some extent by the curing temperature, and different brass powder contents also affected the impact resistance. For the self-repairing decorative coating with 3% brass powder, the impact resistance remained constant at 2 kg·cm regardless of curing temperature. For the coating with 6% brass powder, the impact resistance was 2 kg·cm when the curing temperature ranged from 30 °C to 50 °C. When the curing temperature reached 55 °C, it increased to 3 kg·cm. For the coating with 9% brass powder, the impact resistance remained at 2 kg·cm within the 30–45 °C range. When the curing temperature reached 50 °C, the impact resistance increased to 3 kg·cm. It can be concluded that, for the three water-based coatings with different brass powder contents, the impact resistance generally increased with increasing curing temperature. Since brass powder is a rigid particulate filler, increasing its content can effectively improve the mechanical properties of the microcapsule coating. The presence of these solid fillers helps the coating better maintain its structural integrity under impact loading. Overall, the self-repairing water-based coating with 9% brass powder exhibited the best impact resistance, followed by the coating with 6% brass powder and the coating with 3% brass powder, respectively.

#### 4.3.4. Analysis of Coating Adhesion

The changes in adhesion of self-repairing decorative coatings with different brass powder contents on the surface of the Basswood as a function of curing temperature are shown in Table 13. For the self-repairing decorative coating with 3% brass powder, the adhesion grade was 2 when the curing temperature ranged from 30 °C to 45 °C. With further increases in curing temperature, the adhesion improved significantly, reaching grade 1. For the coating with 6% brass powder, the adhesion grade remained at 2 within the temperature range of 30–55 °C, and improved to grade 1 when the curing temperature increased to 60 °C. For the coating containing 9% brass powder, the adhesion grade was relatively poor at 30 °C (grade 3). As the curing temperature increased from 35 °C to 60 °C, the adhesion improved to grade 2. Overall, adhesion showed a positive correlation with curing temperature for all three coatings; higher curing temperatures resulted in higher adhesion grades and improved bonding performance. An increase in the brass powder content in the coating tended to reduce coating adhesion. This is because the brass powder particles disrupted the compact internal structure of the coating, thereby weakening its cohesion. As a result, the interfacial bonding between the coating and the wooden substrate was adversely affected, leading to reduced adhesion performance.

#### 4.3.5. Analysis of Coating Roughness

The changes in roughness of self-repairing decorative coatings with different brass powder contents on the surface of the Basswood as a function of curing temperature are shown in Table 14. With increasing curing temperature, the surface roughness of the coatings decreased significantly. For the coating containing 3% brass powder, the roughness decreased from 3.848 μm to 1.125 μm. For the 6% brass powder coating, it decreased from 5.115 μm to 1.244 μm, while for the 9% brass powder coating, it decreased from 5.506 μm to 1.398 μm. The increase in temperature reduced the roughness of the self-repairing water-based coatings. This behavior can be attributed to low-temperature curing, which may lead to defects such as uneven resin film formation and trapped air voids, thereby increasing surface roughness. Meanwhile, the brass powder content had a pronounced effect on surface roughness. The coating with 3% brass powder exhibited the lowest roughness and the smoothest surface, followed by the coating with 6% brass powder, while the coating with 9% brass powder showed the highest roughness. This is because the addition of solid brass powder destroyed the uniform compactness of the coating surface, increasing the roughness of the coating surface.

#### 4.3.6. Analysis of Aging Resistance of Coatings

(1) High-Temperature Accelerated Aging

**Figure 16:**
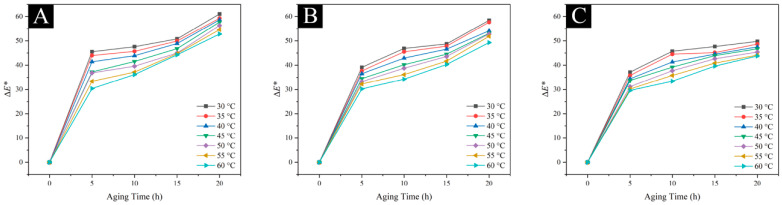
Effect of high-temperature accelerated aging on the chromaticity variation of self-repairing brass powder–water-based acrylic coatings prepared at different curing temperatures: (**A**) 3%, (**B**) 6%, (**C**) 9%.

After the high-temperature accelerated aging test, the changes in ΔE* values of self-repairing brass powder–water-based acrylic decorative coatings on the Basswood surfaces cured under different temperatures are shown in Figure 16. For self-repairing decorative coatings with a certain brass powder content, higher curing temperatures led to smaller chromaticity changes after aging, indicating improved color stability under thermal aging conditions. For the decorative coating with 3% brass powder, the ΔE* value after 20 h of high-temperature accelerated aging was 61.07 for the sample cured at 30 °C, whereas it decreased to 52.82 for the sample cured at 60 °C. For the decorative coating with 6% brass powder, the ΔE* value decreased from 58.49 (30 °C curing) to 49.27 (60 °C curing). Similarly, for the decorative coating with 9% brass powder, the ΔE* value decreased from 49.75 to 43.74 under the same curing conditions.

This can be attributed to the fact that an appropriate increase in curing temperature enhances the crosslinking degree of resin molecules within the coating, thereby promoting the formation of a more compact and stable network structure. As a result, the coating is better able to maintain its structural integrity and performance under high-temperature aging conditions. In addition, it can be observed that, at the same curing temperature, the degree of discoloration of the coatings gradually decreased with increasing brass powder content after high-temperature aging. This suggests that higher filler loading contributes to improved color stability under thermal aging.

(2) UV Aging Resistance

**Figure 17:**
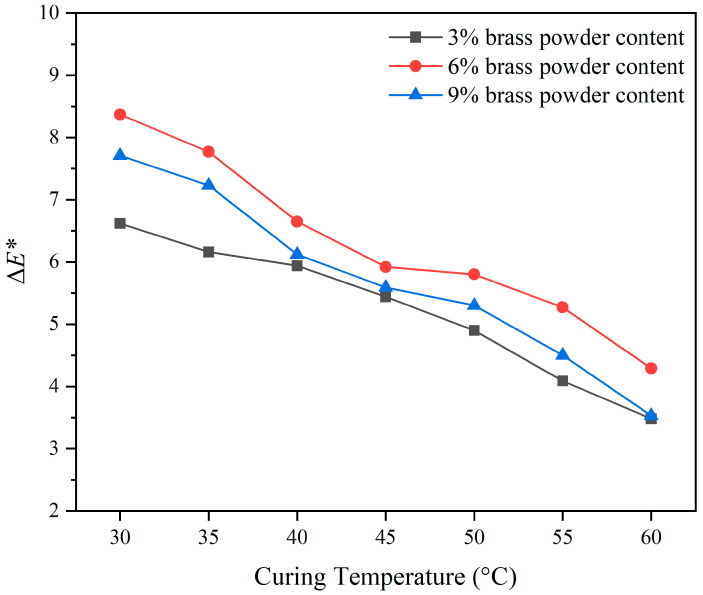
Chromaticity variation of decorative coatings with different brass powder contents, after the UV aging test.

After three cycles of UV aging tests, the changes in ΔE* values of self-repairing brass powder–water-based acrylic decorative coatings on the Basswood surfaces cured at different temperatures are shown in Figure 17. With increasing curing temperature, the ability of the coatings to maintain their original color after UV exposure was gradually enhanced, resulting in lower ΔE* values after aging. For the self-repairing decorative coating with 3% brass powder, the ΔE* value after three UV aging cycles decreased from 6.62 for the sample cured at 30 °C to 3.48 for the sample cured at 60 °C. Similarly, for the coating containing 6% brass powder, the ΔE* value decreased from 8.37 to 4.29 as the curing temperature increased from 30 °C to 60 °C. For the coating containing 9% brass powder, the ΔE* value after three UV aging cycles decreased from 7.71 for the sample cured at 30 °C to 3.53 for the sample cured at 60 °C. Overall, 60 °C was identified as the optimal curing temperature for the self-repairing brass powder–water-based acrylic decorative coating system. Curing at this temperature promoted the formation of a more stable coating structure, which improved the resistance of both the brass powder and acrylic matrix to oxidation and consequently reduced the discoloration rate during UV aging. Furthermore, Figure 17 shows that, at the same curing temperature, the ΔE* values of the coatings gradually decreased with increasing brass powder content after three UV aging cycles, indicating enhanced color stability with higher brass powder loading.

#### 4.3.7. Analysis of Cold Liquid Resistance of Coatings

(1) Liquid Resistance gloss

After the liquid resistance test, the changes in gloss of self-repairing brass powder–water-based acrylic decorative coatings on the Basswood surfaces cured at different temperatures are shown in Table S9. The results showed that the gloss values of all coatings decreased after exposure to the test liquids compared with those before the liquid resistance test. However, the gloss of the coatings remained positively correlated with the curing temperature. Among the four test liquids, coffee and dishwashing detergent had the greatest impact on the gloss of the brass powder coatings, while citric acid and ethanol had a smaller impact on the gloss of the brass powder coatings.

(2) Change of Liquid Resistance Chromaticity

After the liquid resistance test, the chromaticity changes of self-repairing brass powder–water-based acrylic decorative coatings on the Basswood surfaces cured at different temperatures are shown in Table S10. The results indicated that the change in chromaticity after liquid exposure was negatively correlated with the curing temperature; that is, higher curing temperatures resulted in smaller color differences. This behavior can be attributed to the fact that an appropriate curing temperature promotes more complete film formation and produces a more uniform and compact coating structure [42]. The denser coating structure reduces the penetration of test liquids into the coating, thereby minimizing their influence on the coating color. In addition, increasing the brass powder content enhanced the influence of the test liquids on coating color, resulting in greater chromaticity changes after liquid resistance testing. Among them, the water-based acrylic coating containing 3% brass powder and cured at 60 °C exhibited the smallest chromaticity changes after liquid resistance testing, with ΔE* values of 2.11, 2.11, 7.30, and 4.16 after exposure to citric acid, ethanol, dishwashing detergent, and coffee, respectively. Among the four test liquids, coffee and dishwashing detergent caused relatively larger color changes in the brass powder coatings, whereas citric acid and ethanol had weaker effects. For the coating containing 9% brass powder cured at 30 °C, the ΔE* values after coffee and dishwashing detergent exposure reached 12.10 and 9.56, respectively, which were the highest values among all tested samples. However, citric acid and ethanol had a smaller impact on the gloss of the brass powder coatings. In contrast, for the coating containing 9% brass powder cured at 60 °C, the ΔE* values after coffee and dishwashing detergent exposure decreased to 5.70 and 7.74, respectively. The increased color variation caused by coffee and dishwashing detergent may be associated with their complex compositions, including organic pigments and surfactants, which can interact with the coating surface and promote liquid penetration.

(3) Change of Liquid resistance grade

The liquid resistance classification results of self-repairing brass powder–water-based acrylic decorative coatings applied on Basswood surfaces and cured at different temperatures are shown in Table 15. The effects of ethanol and dishwashing detergent on the coatings were less dependent on the curing temperature. Regardless of the curing temperature and brass powder content, all coatings exhibited grade 1 resistance to ethanol and grade 2 resistance to dishwashing detergent. When the curing temperature ranged from 30 to 55 °C, the coatings exhibited grade 2 resistance to coffee, with slight discoloration observed on the coating surface. When the curing temperature rose to 60 °C, the liquid resistance grade against coffee improved to grade 1. Overall, increasing the curing temperature enhanced the liquid resistance of the self-repairing decorative coatings. This can be attributed to the formation of a more complete crosslinked network structure of the water-based acrylic coating at an appropriate curing temperature. The enhanced curing process helps reduce coating defects and voids, resulting in a more uniform and compact coating structure. Therefore, curing at 60 °C improved the liquid resistance of the coating. The coatings containing 6% and 9% brass powder exhibited similar liquid resistance behavior. When the curing temperature ranged from 30 to 35 °C, their resistance to citric acid was classified as grade 2. In contrast, the coating containing 3% brass powder exhibited grade 1 resistance to citric acid under the same conditions. This difference may be attributed to the susceptibility of brass powder to react with citric acid, causing surface discoloration [43]. A higher brass powder content provides more reactive sites, thereby intensifying the color change after acid exposure.

In summary, the curing temperature of 60 °C was identified as the optimum condition. From a chemical perspective, increasing the curing temperature enhanced polymer chain mobility and facilitated the crosslinking reaction of the water-based acrylic resin, leading to the formation of a more compact and uniform coating film. This improved film structure reduced residual volatile components and minimized microstructural defects, thereby enhancing optical properties, including gloss, reflectance, and color uniformity. In contrast, insufficient curing at lower temperatures resulted in incomplete film formation and the retention of residual components, which negatively affected the optical performance of the coatings. From a practical application perspective, 60 °C provided a suitable balance between curing efficiency and energy consumption while avoiding potential thermal damage to the wood substrate and microcapsule structures. Therefore, 60 °C was selected as the optimal curing temperature based on both the underlying curing mechanism and practical application considerations.

#### 4.3.8. Analysis of Self-Repairing Performance of Coatings

**Figure 18:**
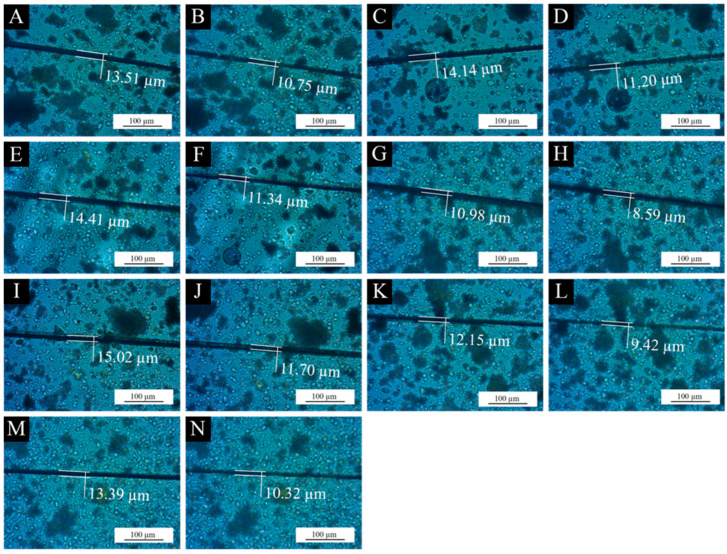
Scratch repair of self-repairing decorative coatings adding 3% brass powder: (**A**) sample 10 before repair, (**B**) sample 10 after repair, (**C**) sample 11 before repair, (**D**) sample 11 after repair, (**E**) sample 12 before repair, (**F**) sample 12 after repair, (**G**) sample 13 before repair, (**H**) sample 13 after repair, (**I**) sample 14 before repair, (**J**) sample14 after repair, (**K**) sample 15 before repair, (**L**) sample 15 after repair, (**M**) sample 16 before repair, (**N**) sample 16 after repair.

**Figure 19:**
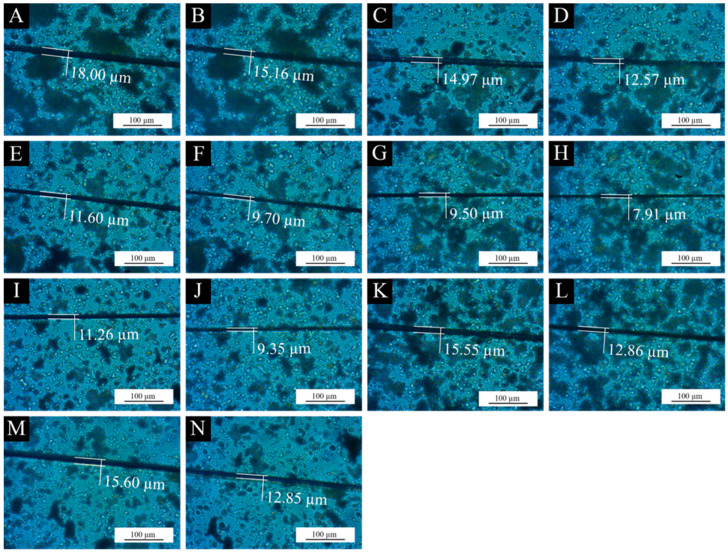
Scratch repair of self-repairing decorative coatings adding 6% brass powder: (**A**) sample 17 before repair, (**B**) sample 17 after repair, (**C**) sample 18 before repair, (**D**) sample 18 after repair, (**E**) sample 19 before repair, (**F**) sample 19 after repair, (**G**) sample 20 before repair, (**H**) sample 20 after repair, (**I**) sample 21 before repair, (**J**) sample 21 after repair, (**K**) sample 22 before repair, (**L**) sample 22 after repair, (**M**) sample 23 before repair, (**N**) sample 23 after repair.

**Figure 20:**
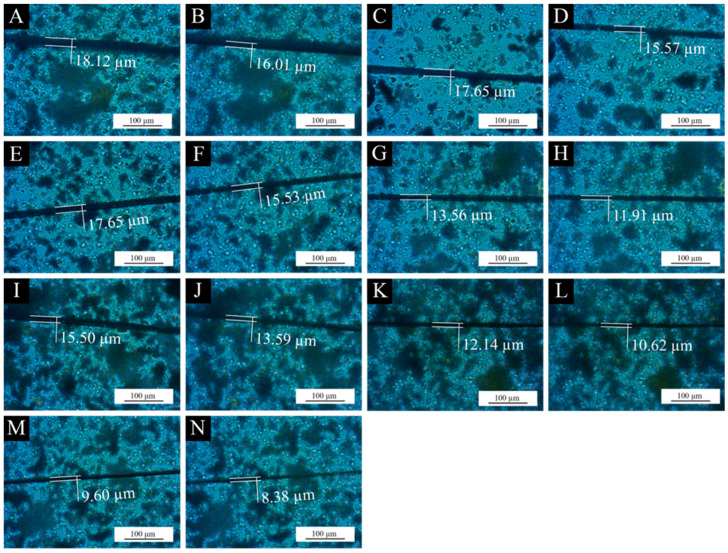
Scratch repair of self-repairing decorative coatings adding 9% brass powder: (**A**) sample 24 before repair, (**B**) sample 24 after repair, (**C**) sample 25 before repair, (**D**) sample 25 after repair, (**E**) sample 26 before repair, (**F**) sample 26 after repair, (**G**) sample 27 before repair, (**H**) sample 27 after repair, (**I**) sample 28 before repair, (**J**) sample 28 after repair, (**K**) sample 29 before repair, (**L**) sample 29 after repair, (**M**) sample 30 before repair, (**N**) sample 30 after repair.

The scratch repair performance of self-repairing brass powder–water-based acrylic decorative coatings on Basswood surfaces prepared at different curing temperatures is shown in Figure 18, Figure 19 and Figure 20 and Table 16. For the self-repairing coating containing 3% brass powder, the repair rate increased from 20.43% to 22.93% as the curing temperature increased, corresponding to an improvement of 2.50%. Similarly, the repair rate of the coatings containing 6% and 9% brass powder increased from 15.78% to 17.63% and from 11.64% to 12.71%, respectively, with increases of 1.85% and 1.07%. Overall, increasing the curing temperature enhanced the self-repairing performance of the coatings. Meanwhile, the brass powder content also influenced the self-repairing performance of the coatings. The coating containing 3% brass powder exhibited the highest repair efficiency. This may be attributed to the fact that excessive brass powder loading disrupted the uniformity and compactness of the coating structure, resulting in localized stress concentration within the coating. Under mechanical stress, these stress concentrations may induce premature rupture of microcapsules, leading to the early release and consumption of healing agents and reducing the self-repairing capability of the coating. Meanwhile, the encapsulation efficiency of the microcapsules also played an important role in determining the repair performance. Microcapsules with higher encapsulation efficiency could store more healing agent and release sufficient repair material upon rupture, thereby facilitating crack filling and improving the repair efficiency. In addition, excessive brass powder could hinder the flow of the released healing agent into damaged regions during the repair process, thereby limiting subsequent curing and reducing the self-repairing performance of the coating.

The self-repairing behavior of the coating was evaluated based on scratch-width reduction; however, the repairing process was not merely a geometric recovery phenomenon but was closely associated with a microcapsule-triggered release mechanism. When the coating was subjected to mechanical damage, crack propagation generated local stress concentration at the crack tip, causing the rupture of melamine-formaldehyde microcapsule shells embedded within the coating matrix once the stress exceeded their mechanical strength. Subsequently, the encapsulated water-based acrylic primer was released into the damaged region. The released primer then underwent solvent evaporation and in situ film formation, forming a continuous polymer layer that filled and bridged the crack, thereby restoring the structural integrity of the coating. It was noted that the observed repair effect was unlikely to be dominated by viscoelastic recovery of the polymer matrix. In general, viscoelastic relaxation in polymer coatings led to limited and reversible deformation, such as elastic recovery and partial closure of shallow scratches, rather than complete crack filling or structural reconstruction, while the significant and localized crack repair observed in this system was more consistent with the release of the encapsulated repairing agent. Therefore, the self-repairing performance was mainly attributed to microcapsule rupture and subsequent release of the water-based acrylic primer.

To better evaluate the performance of the present self-repairing coating, a comparison with representative self-repairing systems reported in the literature was conducted. Epoxy-based self-repairing coatings, typically based on microcapsule-encapsulated curing agents, exhibited high mechanical strength but were often limited by the intrinsic brittleness of the crosslinked network, resulting in reduced flexibility and crack resistance under deformation. For instance, Ghazali et al. [44] reported an elevated-temperature cured epoxy system with dual microcapsules (DGEBA and mercaptan), which showed partial recovery of fracture toughness after thermal activation at 70 °C. Polyurea-based systems were reported to exhibit excellent toughness and rapid repairing behavior. For example, Mu et al. [45] developed a dual-microcapsule system based on diamine and IPDI, forming polyurea-shelled microcapsules that enabled effective self-repairing in polymer matrices; however, their synthesis and processing were relatively complex and costly.

Compared with the coating system reported by Chang et al. [21], as summarized in Table 17, where both topcoat microcapsules and modified brass powder were incorporated into the topcoat and cured at room temperature (approximately 25 °C), the coating system developed in this study exhibited a higher self-repairing efficiency. In the present work, primer microcapsules and modified brass powder were incorporated into the primer layer, and the coating was cured at 30 °C. The coating containing 3% brass powder and 3% microcapsules with a core–wall ratio of 0.58:1 (Sample 10) achieved a repair rate (*D_H_*) of 20.43%, which was higher than those reported by Chang et al. [21] for coatings containing the same brass powder content and microcapsule content but different core–wall ratios, including 19.62% (core–wall ratio of 0.58:1, Sample 13), 17.41% (core–wall ratio of 0.67:1, Sample 21), and 19.55% (core–wall ratio of 0.75:1, Sample 29). This is because of the different locations of microcapsule incorporation. For effective self-repair, microcapsules need to be uniformly embedded within the coating matrix and rupture upon external damage to release healing agents. The relatively strong adhesion and compact structure of the primer layer may provide better fixation and stress transfer for the microcapsules, facilitating the triggered release of healing agents and improving the repair efficiency. Compared with these systems, the coating developed in this work demonstrated promising self-repairing capability and satisfactory overall performance, which can be attributed to the effective encapsulation of healing agents and the controlled release behavior of the microcapsules. Therefore, the proposed system achieved a balanced combination of self-repairing efficiency, processing feasibility, and water-based characteristics.

#### 4.3.9. Analysis of Microstructure and Infrared Spectroscopy of Coatings

(1) Microscopic Morphology of Coatings

**Figure 21:**
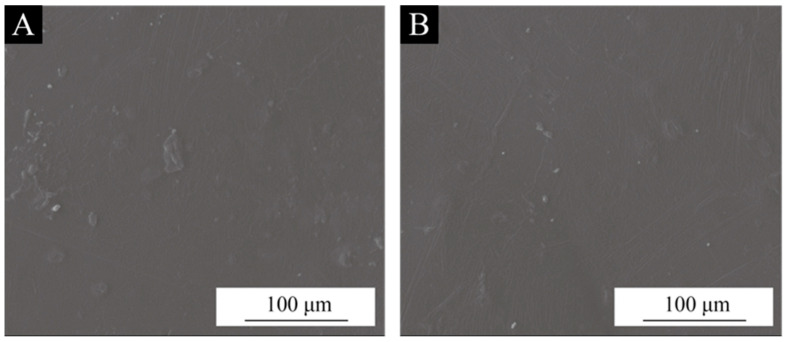
SEM of the 3% microcapsules-3% brass powder–water-based acrylic decorative coatings obtained by curing at different temperatures: (**A**) 30 °C (sample 10), (**B**) 60 °C (sample 16).

Based on the above results, the coating formulation containing 3% modified brass powder and 3% microcapsules (core–wall ratio of 0.58:1) in the water-based acrylic primer and cured at 60 °C (Sample 16) exhibited the best overall performance. Compared with the optimal sample (Sample 13) reported by Chang et al. [21], Sample 16 showed improved optical, mechanical, and functional properties. Specifically, the gloss increased to 34.1 GU, while the color uniformity was enhanced with a Δ*E* value of 0.00. The visible light reflectance (R) and transmittance reached 0.6038 and 76.20%, respectively, and the dominant wavelength was 589.79 nm. In terms of mechanical properties, the coating exhibited a hardness of HB, an impact resistance of 2 kg·cm, and grade 1 adhesion. In addition, the coating surface showed improved smoothness with a roughness of 1.125 μm, accompanied by enhanced aging resistance, liquid resistance, and self-repairing performance (repair rate: 22.93%). Therefore, the surface morphology of Sample 16 was further characterized, as shown in Figure 21B. Compared with the coating prepared at 30 °C using the same formulation (Sample 10, Figure 21A), the coating cured at 60 °C exhibited a flatter and smoother surface morphology. This observation was consistent with the roughness results (Table 14), confirming the improved surface quality obtained at the higher curing temperature.

(2) Coating Analysis by Infrared Spectroscopy

**Figure 22:**
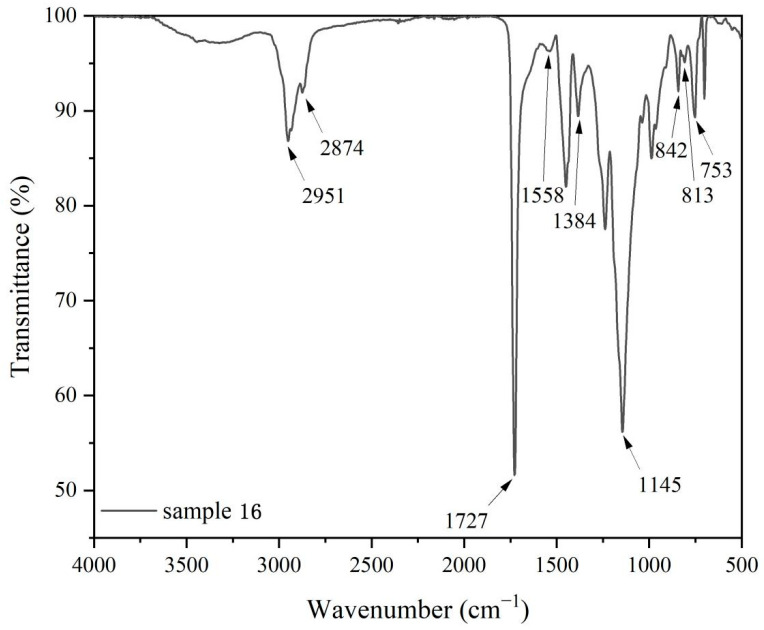
FT-IR spectra of the coating obtained by curing at 60 °C (sample 16).

Figure 22 shows the FTIR spectrum of the microcapsule–brass powder–water-based acrylic coating cured at 60 °C (sample 16). A characteristic peak of methylene groups was observed at 2874 cm^−1^, and the band at 2951 cm^−1^ was attributed to the C–H stretching vibration of –CH_3_ in the acrylic resin matrix. The strong absorption peak at 1727 cm^−1^ corresponded to the C=O stretching vibration of ester groups in the water-based acrylic resin. A weak band appearing at 1558 cm^−1^ could be attributed to N–H bending vibration and C–N stretching of the triazine ring in the melamine resin shell, indicating the presence of the microcapsule structure. The peak at 1384 cm^−1^ was assigned to CH_3_ deformation vibration and N–H related vibrations. A characteristic peak of the triazine ring in melamine resin was detected at 813 cm^−1^, further confirming the existence of the microcapsule shell. The bands at 842 cm^−1^ and 753 cm^−1^ were attributed to Si–C stretching vibrations of KH570-modified brass powder fillers, while the peak at 1144 cm^−1^ was associated with Si–O–M (M = Si, Cu, Zn) asymmetric stretching vibrations.

## 5. Conclusions

A self-repairing brass powder–water-based acrylic decorative coating for Basswood was successfully fabricated by incorporating melamine resin-coated water-based primer microcapsules and KH570-modified brass powder into the primer layer through a “three coats of primer and two coats of topcoat” process. Orthogonal analysis demonstrated that curing temperature was the dominant factor affecting the optical and self-repairing properties of the coating, whereas brass powder content had a greater influence on mechanical performance. Increasing the curing temperature promoted resin crosslinking and improved film formation, thereby enhancing the optical appearance, mechanical properties, aging resistance, liquid resistance, and self-repairing capability of the coatings. Among all tested formulations, the coating containing 3% brass powder and 3% primer microcapsules with a core–wall ratio of 0.58:1, cured at 60 °C, exhibited optimal comprehensive performance. This optimized coating achieved a gloss of 34.1 GU, a Δ*E* value of 0.00, a visible light transmittance of 76.20%, a hardness of HB, grade 1 adhesion, a surface roughness of 1.125 μm, and a self-repairing efficiency of 22.93%, together with satisfactory aging and liquid resistance. The enhanced performance was attributed to the combined effects of optimized curing conditions and the effective incorporation of brass powder and microcapsules into the primer layer. The optimized coating structure improved resin network formation while maintaining the functionality of microcapsules, enabling a balance between decorative performance, mechanical durability, and self-repairing capability. This work provides an effective strategy for developing multifunctional water-based protective coatings for wood and bamboo substrates.

## Figures and Tables

**Table 1:** Table of test materials.

Test Materials	Molecular Formula	Purity	Manufacturer
Brass powder	-	AR	Nangong Xindun Alloy Welding Material Spraying Co., Ltd., Xingtai, China
γ-methacryloxypropyltrimethoxysilane (KH570)	C_10_H_20_O_5_Si	AR	Hangzhou Jessica Chemicals Co., Ltd., Hangzhou, China
Dulux water-based acrylic primer and topcoat	-	AR	Dulux Coatings Co., Ltd., Shanghai, China
Formaldehyde solution	CH_2_O	37%	Xi’an Tianmao Chemical Co., Ltd., Xi’an, China
Melamine	C_3_H_6_N_6_	AR	Shandong Yousuo Chemical Technology Co., Ltd., Linyi, China
Triethanolamine	C_6_H_15_NO_3_	AR	Guangzhou Jiale Chemical Co., Ltd., Guangzhou, China
Sodium Dodecyl Benzene Sulfonate (SDBS)	C_18_H_29_NaO_3_S	AR	Tianjin Beichen Fangzheng Reagent Factory, Tianjin, China
Citric acid monohydrate	C_6_H_10_O_8_	AR	Suzhou Changjiu Chemical Technology Co., Ltd., Suzhou, China
Anhydrous ethanol	C_2_H_6_O	AR	Wuxi Jingke Chemical Co., Ltd., Wuxi, China
Basswood boards	-	-	Beijing Yidimei Model Co., Ltd., Beijing, China
Dishwashing detergent	-	-	Lishui Diaopai Chemical Co., Ltd., Lishui, China
Coffee	-	-	UCC Ueshima Coffee Co., Ltd., Kobe, Japan

**Table 2:** List of raw material usage for microcapsules of water-based primer coated with melamine resin with different core-wall ratios.

Core-Wall Ratio	Melamine (g)	37% Formaldehyde (g)	Deionized Water (g)
0.58:1	10.06	19.52	50.29
0.67:1	8.71	16.81	43.53
0.75:1	7.78	15.02	38.89

**Table 3:** Single-factor test schedule.

Sample	Content of Brass Powder (g)	Microcapsule Weight (g)	Primer Weight (g)	Topcoat Weight (g)	Curing Temperature (°C)
10	0.06	0.06	1.88	2.00	30
11	0.06	0.06	1.88	2.00	35
12	0.06	0.06	1.88	2.00	40
13	0.06	0.06	1.88	2.00	45
14	0.06	0.06	1.88	2.00	50
15	0.06	0.06	1.88	2.00	55
16	0.06	0.06	1.88	2.00	60
17	0.12	0.06	1.82	2.00	30
18	0.12	0.06	1.82	2.00	35
19	0.12	0.06	1.82	2.00	40
20	0.12	0.06	1.82	2.00	45
21	0.12	0.06	1.82	2.00	50
22	0.12	0.06	1.82	2.00	55
23	0.12	0.06	1.82	2.00	60
24	0.18	0.06	1.76	2.00	30
25	0.18	0.06	1.76	2.00	35
26	0.18	0.06	1.76	2.00	40
27	0.18	0.06	1.76	2.00	45
28	0.18	0.06	1.76	2.00	50
29	0.18	0.06	1.76	2.00	55
30	0.18	0.06	1.76	2.00	60

**Table 4:** Grading table of cold liquid resistance of the coating.

Grade	Explanation
1	No change; The test area cannot be distinguished from the adjacent area.
2	Slight changes: Only when the light source is projected onto the test surface and reflected into the observer’s eyes can the test area be distinguished from the adjacent area, such as fading, discoloration, and color change. The surface structure of the test remains unchanged, such as expansion, fiber protrusion, cracking, or bubbling.
3	Moderate change: It can be seen in several directions, and the test area can be distinguished from the adjacent area, such as fading, discoloration, and color change. There was no change in the surface structure of the test, such as expansion, fiber protrusion, cracking, or bubbling.
4	Obvious changes: Visible in all visible directions, the test area can be clearly distinguished from the adjacent areas, such as fading, discoloration, and color change; and/or there are slight changes in the surface structure of the test, such as expansion, fiber protrusion, cracking, or bubbling.
5	Serious change: The surface structure of the test has changed significantly; and/or fading, discoloration, and change color; and/or all or part of the surface material is removed; and/or the filter paper is stuck to the surface.

**Table 5:** Encapsulation efficiency of melamine resin-coated water-based primer microcapsules with different core–wall ratios.

Microcapsule Sample	Core-Wall Ratio	Encapsulation Efficiency (%)
1	0.58:1	54.3
2	0.67:1	41.7
3	0.75:1	36.6

**Table 6:** Visual analysis of gloss.

Sample	Content of Brass Powder (%)	Core-Wall Ratio	Microcapsule Content (%)	Curing Temperature (°C)	Gloss (GU)
1	3	0.58:1	3	25	37.8 ± 0.71
2	3	0.67:1	6	40	24.9 ± 0.65
3	3	0.75:1	9	60	44.7 ± 0.80
4	6	0.58:1	6	60	38.2 ± 0.57
5	6	0.67:1	9	25	24.5 ± 0.43
6	6	0.75:1	3	40	29.5 ± 0.73
7	9	0.58:1	9	40	28.3 ± 0.28
8	9	0.67:1	3	60	54.7 ± 1.04
9	9	0.75:1	6	25	25.6 ± 0.62
Mean 1 ± Standard Deviation 1	35.800 ± 10.050	34.767 ± 5.604	40.667 ± 12.842	29.300 ± 7.382	
Mean 2 ± Standard Deviation 2	30.733 ± 6.933	34.700 ± 17.322	29.567 ± 7.485	27.567 ± 2.386	
Mean 3 ± Standard Deviation 3	36.200 ± 16.078	33.267 ± 10.092	32.500 ± 10.735	45.867 ± 8.312	
Extreme difference	5.467	1.500	11.100	18.300	

**Table 7:** Analysis of variance of gloss.

Factor	Sum of Squares (SS)	Degree of Freedom	F Ratio	F-Critical	*p*-Value
Content of brass powder	55.723	2	12.929	19.000	0.072
Core-wall ratio	4.308	2	1.000	19.000	0.500
Microcapsule content	198.512	2	46.059	19.000	0.021 *
Curing temperature	612.359	2	142.079	19.000	0.007 **
Error	4.31	2			

* *p* < 0.05 ** *p* < 0.01.

**Table 8:** Visual analysis of elongation at break.

Sample	Content of Brass Powder (%)	Core-Wall Ratio	Microcapsule Content (%)	Curing Temperature (°C)	Elongation at Break (%)
1	3	0.58:1	3	25	5.09 ± 0.12
2	3	0.67:1	6	40	6.25 ± 0.11
3	3	0.75:1	9	60	5.64 ± 0.18
4	6	0.58:1	6	60	3.08 ± 0.08
5	6	0.67:1	9	25	3.78 ± 0.09
6	6	0.75:1	3	40	4.47 ± 0.11
7	9	0.58:1	9	40	5.66 ± 0.17
8	9	0.67:1	3	60	5.05 ± 0.14
9	9	0.75:1	6	25	4.76 ± 0.10
Mean 1 ± Standard Deviation 1	5.660 ± 0.580	4.610 ± 1.355	4.870 ± 0.347	4.543 ±0.681	
Mean 2 ± Standard Deviation 2	3.777 ± 0.695	5.027 ± 1.235	4.697 ± 1.586	5.460 ± 0.907	
Mean 3 ± Standard Deviation 3	5.157 ± 0.459	4.957 ± 0.609	5.027 ± 1.080	4.590 ± 1.341	
Extreme difference	1.883	0.417	0.330	0.917	

**Table 9:** Analysis of variance of elongation at break.

Factor	Sum of Squares (SS)	Degree of Freedom	F Ratio	F-Critical	*p*-Value
Content of brass powder	5.705	2	35.000	19.000	0.028 *
Core-wall ratio	0.299	2	1.834	19.000	0.353
Microcapsule content	0.163	2	1.000	19.000	0.500
Curing temperature	1.599	2	9.810	19.000	0.093
Error	0.16	2			

* *p* < 0.05.

**Table 10:** Transmittance of decorative coatings with different brass powder contents.

Curing Temperature (°C)	Transmittance (%)		
	3%	6%	9%
30	59.69 ± 1.22	58.51 ± 1.09	47.64 ± 0.82
35	66.01 ± 0.82	61.95 ± 1.38	51.56 ± 1.94
40	66.66 ± 1.24	68.90 ± 0.83	60.36 ± 0.96
45	67.41 ± 1.00	64.00 ± 1.41	54.05 ± 0.82
50	71.84 ± 1.32	65.40 ± 0.99	59.76 ± 1.45
55	72.80 ± 1.40	70.89 ± 1.34	60.43 ± 1.02
60	76.20 ± 1.32	70.98 ± 0.82	61.27 ± 0.90

**Table 11:** Effect of curing temperature on the hardness of coatings with different brass powder contents.

Curing Temperature (°C)	Hardness		
	3%	6%	9%
30	B	HB	HB
35	B	HB	HB
40	B	HB	HB
45	B	HB	HB
50	B	HB	H
55	B	H	H
60	HB	H	H

**Table 12:** Effect of curing temperature on the impact resistance of coatings with different brass powder contents.

Curing Temperature (°C)	Impact Resistance (kg·cm)		
	3%	6%	9%
30	2	2	2
35	2	2	2
40	2	2	2
45	2	2	2
50	2	2	3
55	2	3	3
60	2	3	3

**Table 13:** Effect of curing temperature on the adhesion of decorative coatings with different brass powder contents.

Curing Temperature (°C)	Adhesion (Grade)		
	3%	6%	9%
30	2	2	3
35	2	2	2
40	2	2	2
45	2	2	2
50	1	2	2
55	1	2	2
60	1	1	2

**Table 14:** Effect of curing temperature on the roughness of coatings with different brass powder contents.

Curing Temperature (°C)	Roughness (µm)		
	3%	6%	9%
30	3.848 ± 0.123	5.115 ± 0.152	5.506 ± 0.203
35	3.671 ± 0.095	3.737 ± 0.102	4.479 ± 0.127
40	2.059 ± 0.063	2.464 ± 0.045	2.764 ± 0.082
45	1.962 ± 0.044	2.099 ± 0.077	2.257 ± 0.054
50	1.480 ± 0.043	1.625 ± 0.039	1.765 ± 0.039
55	1.165 ± 0.025	1.453 ± 0.041	1.472 ± 0.037
60	1.125 ± 0.025	1.244 ± 0.033	1.398 ± 0.011

**Table 15:** Effect of curing temperature on the grade of liquid resistance of self-repairing brass powder–water-based acrylic decorative coating.

Sample	Content of Brass Powder (%)	Curing Temperature (°C)	Grade of Liquid Resistance (Grade)			
			Citric Acid	Ethanol	Dishwashing Detergent	Coffee
10	3	30	1	1	2	2
11		35	1	1	2	2
12		40	1	1	2	2
13		45	1	1	2	2
14		50	1	1	2	2
15		55	1	1	2	2
16		60	1	1	2	1
17	6	30	2	1	2	2
18		35	2	1	2	2
19		40	1	1	2	2
20		45	1	1	2	2
21		50	1	1	2	2
22		55	1	1	2	2
23		60	1	1	2	1
24	9	30	2	1	2	2
25		35	2	1	2	2
26		40	1	1	2	2
27		45	1	1	2	2
28		50	1	1	2	2
29		55	1	1	2	2
30		60	1	1	2	1

**Table 16:** Effect of curing temperature on scratch repair of self-repairing decorative coatings, adding different brass powder contents.

Curing Temperature (°C)	*D_H_* (%)		
	3%	6%	9%
30	20.43 ± 0.41	15.78 ± 0.57	11.64 ± 0.10
35	20.79 ± 0.53	16.03 ± 0.25	11.78 ± 0.32
40	21.30 ± 0.58	16.38 ± 0.37	12.01 ± 0.38
45	21.77 ± 0.21	16.74 ± 0.59	12.17 ± 0.21
50	22.10 ± 0.63	16.96 ± 0.13	12.32 ± 0.33
55	22.47 ± 0.61	17.30 ± 0.51	12.52 ± 0.38
60	22.93 ± 0.73	17.63 ± 0.50	12.71 ± 0.28

**Table 17:** Comparison of self-repairing efficiency (*D_H_*) between this work and previous literature.

Sample	Coated Core Material	Core–Wall Ratio	Brass Powder Content (%)	Microcapsule Content (%)	Curing Temperature (°C)	Self-Repairing Efficiency (*D_H_*, %)
Sample 13 from reference [21]	Topcoat	0.58:1	3	3	room temperature (approximately 25)	19.62
Sample 21 from reference [21]	Topcoat	0.67:1	3	3	room temperature (approximately 25)	17.41
Sample 29 from reference [21]	Topcoat	0.75:1	3	3	room temperature (approximately 25)	19.55
Sample 10 from this work	Primer	0.58:1	3	3	30	20.43
Sample 16 from this work	Primer	0.58:1	3	3	60	22.93

## Data Availability

The original contributions presented in this study are included in the article/Supplementary Materials. Further inquiries can be directed to the corresponding author.

## Supplementary Materials

The following supporting information can be downloaded at: https://www.mdpi.com/article/10.3390/polym18141773/s1, Table S1: Orthogonal test factors and levels; Table S2: Orthogonal test schedule; Table S3: List of raw material consumption in the orthogonal test; Table S4: Gloss of brass powder coating in the orthogonal test; Table S5: Effect of curing temperature on gloss of decorative coatings with different brass powder contents; Table S6: Effect of curing temperature on Δ*E* of decorative coatings with different brass powder contents; Table S7: Effect of curing temperature on R value of decorative coatings with different brass powder contents; Table S8: Effect of curing temperature on the main wavelength of coatings with different brass powder contents; Table S9: Effect of curing temperature on liquid resistance gloss of self-repairing brass powder-water-based acrylic decorative coatings; Table S10: Effect of curing temperature on liquid resistance chromaticity variation of self-repairing brass powder-water-based acrylic decorative coatings.
