# Supplementary material for: Preparation and Performance Characterization of Melamine Resin-Coated Water-Based Primer Microcapsule–Brass Powder–Water-Based Acrylic Coating
Source: Polymers (Basel). 2026 Jul 20;18(14):1773. doi: 10.3390/polym18141773 (PMC13419264; doi:10.3390/polym18141773)
Supplement: Supplementary file 1 [file polymers-18-01773-s001.zip › polymers-4409341-supplementary.pdf]

Table S1. Orthogonal test factors and levels.

Level	Content of brass powder (%)	Core-wall ratio	Microcapsule content (%)	Curing temperature (°C)
1	3	0.58:1	3	25
2	6	0.67:1	6	40
3	9	0.75:1	9	60

Table S2. Orthogonal test schedule.

Sample	Content of brass powder (%)	Core-wall ratio	Microcapsule content (%)	Curing temperature (°C)
1	3	0.58:1	3	25
2	3	0.67:1	6	40
3	3	0.75:1	9	60
4	6	0.58:1	6	60
5	6	0.67:1	9	25
6	6	0.75:1	3	40
7	9	0.58:1	9	40
8	9	0.67:1	3	60
9	9	0.75:1	6	25

Table S3. List of raw material consumption in the orthogonal test.

Sample	Content of brass powder (g)	Core-wall ratio	Microcapsule weight (g)	Primer weight (g)	Topcoat weight (g)
1	0.06	0.58:1	0.06	1.88	2.00
2	0.06	0.67:1	0.12	1.82	2.00
3	0.06	0.75:1	0.18	1.76	2.00
4	0.12	0.58:1	0.12	1.76	2.00
5	0.12	0.67:1	0.18	1.70	2.00
6	0.12	0.75:1	0.06	1.82	2.00
7	0.18	0.58:1	0.18	1.64	2.00
8	0.18	0.67:1	0.06	1.76	2.00
9	0.18	0.75:1	0.12	1.70	2.00

Table S4. Gloss of brass powder coating in the orthogonal test.

Sample	Gloss (GU)		
	20°	60°	85°
1	9.2±0.2	37.8±0.7	39.4±0.8

2	7.7±0.2	24.9±0.7	23.3±0.6
3	17.3±0.4	44.7±1.0	44.7±0.9
4	8.4±0.2	38.2±1.1	42.6±0.9
5	8.0±0.1	24.5±0.6	20.7±0.5
6	9.0±0.2	29.5±0.7	35.8±0.6
7	7.0±0.2	28.3±0.9	31.6±0.4
8	13.3±0.4	54.7±0.9	51.2±0.9
9	6.6±0.2	25.6±0.5	25.3±0.4

Table S5. Effect of curing temperature on gloss of decorative coatings with different brass powder contents.

Sample	Content of brass Powder (%)	Curing temperature (°C)	Gloss (GU)		
			20°	60°	85°
10		30	4.5±0.1	15.3±0.4	14.6±0.4
11		35	6.0±0.1	19.6±0.4	18.5±0.5
12		40	7.5±0.2	24.9±0.5	24.8±0.7
13	3	45	8.5±0.2	26.3±0.5	27.2±0.5
14		50	9.5±0.1	28.8±0.6	27.9±0.7
15		55	13.2±0.3	33.5±1.1	32.9±0.7
16		60	13.0±0.3	34.1±0.4	35.5±0.7
17		30	3.4±0.1	12.4±0.4	12.1±0.3
18		35	4.8±0.1	16.4±0.4	14.3±0.4
19		40	5.9±0.1	19.9±0.6	19.0±0.3
20	6	45	8.6±0.3	25.7±0.6	26.6±0.5
21		50	9.4±0.2	28.4±0.4	27.0±0.6
22		55	11.0±0.3	30.5±0.7	26.9±0.5
23		60	11.4±0.3	30.9±0.5	33.3±0.4
24		30	3.0±0.1	11.6±0.2	10.8±0.3
25		35	4.0±0.1	14.0±0.2	11.9±0.2
26		40	4.7±0.1	17.1±0.4	15.7±0.3
27	9	45	5.6±0.1	20.6±0.4	25.1±0.4
28		50	6.1±0.1	21.0±0.4	20.5±0.5
29		55	6.7±0.1	21.8±0.5	22.9±0.6
30		60	8.3±0.2	26.7±0.5	25.9±0.6

Table S6. Effect of curing temperature on ΔE of decorative coatings with different brass powder contents.

Sample	Content of brass Powder (%)	Curing temperature (°C)	Color difference (ΔE)

10		30	4.01±0.10
11		35	3.55±0.07
12		40	2.64±0.04
13	3	45	0.00±0.00
14		50	0.00±0.00
15		55	0.00±0.00
16		60	0.00±0.00
17		30	4.39±0.13
18		35	3.11±0.09
19		40	3.11±0.08
20	6	45	0.00±0.00
21		50	0.00±0.00
22		55	0.00±0.00
23		60	0.00±0.00
24		30	9.65±0.22
25		35	9.65±0.28
26		40	8.51±0.21
27	9	45	0.00±0.00
28		50	0.00±0.00
29		55	0.00±0.00
30		60	0.00±0.00

Table S7. Effect of curing temperature on R value of decorative coatings with different brass powder contents.

Curing temperature (°C)	R		
	3%	6%	9%
30	0.4770±0.0128	0.4321±0.0087	0.3751±0.0086
35	0.4775±0.0089	0.4545±0.0104	0.3885±0.0145
40	0.5205±0.0082	0.4663±0.0083	0.3913±0.0055
45	0.5361±0.0119	0.4746±0.0104	0.4090±0.0078
50	0.5370±0.0128	0.4804±0.0168	0.4315±0.0084
55	0.5393±0.0082	0.5123±0.0088	0.4386±0.0146
60	0.6038±0.0124	0.5229±0.0091	0.4493±0.0082

Table S8. Effect of curing temperature on the main wavelength of coatings with different brass powder contents.

Curing temperature (°C)	Main wavelength (nm)		
	3%	6%	9%
30	586.78±10.99	586.81±10.68	586.97±9.92

35	587.46±12.16	586.82±14.09	587.04±11.00
40	587.47±10.43	587.06±9.69	587.16±13.98
45	587.62±14.06	587.54±12.35	587.64±10.28
50	587.76±12.26	587.21±5.72	587.46±13.79
55	588.09±14.22	588.36±9.59	587.65±8.39
60	589.79±6.63	588.45±11.29	588.07±13.77

Table S9. Effect of curing temperature on liquid resistance gloss of self-repairing brass powder-water-based acrylic decorative coatings.

Sample	Content of brass Powder (%)	Curing temperature (°C)	Liquid resistance gloss (GU)			
			Citric acid	Ethanol	Dishwashing detergent	Coffee
10	3	30	10.0±0.3	12.1±0.3	10.7±0.4	8.1±0.2
11		35	10.6±0.3	13.8±0.4	11.8±0.4	9.8±0.1
12		40	16.4±0.5	18.4±0.6	14.1±0.5	10.0±0.2
13		45	17.2±0.5	19.7±0.4	16.5±0.5	10.1±0.3
14		50	17.3±0.3	21.3±0.6	17.0±0.3	10.8±0.2
15		55	18.3±0.6	22.0±0.7	17.7±0.7	13.3±0.2
16		60	18.5±0.4	23.4±0.3	23.3±0.7	20.9±0.4
17	6	30	11.9±0.1	13.5±0.4	9.5±0.2	6.0±0.1
18		35	12.5±0.4	14.4±0.4	13.0±0.3	6.3±0.2
19		40	13.3±0.4	17.4±0.5	14.7±0.5	6.6±0.1
20		45	14.0±0.3	17.8±0.3	15.1±0.4	16.8±0.6
21		50	16.3±0.4	18.2±0.5	18.0±0.3	17.1±0.4
22		55	17.9±0.5	20.9±0.7	18.0±0.4	17.8±0.4
23		60	18.1±0.5	22.7±0.6	19.5±0.6	18.7±0.5
24	9	30	9.8±0.2	12.2±0.3	8.5±0.2	5.8±0.1
25		35	13.0±0.3	13.5±0.4	9.2±0.1	7.9±0.2
26		40	13.6±0.4	14.8±0.2	15.2±0.3	8.0±0.1
27		45	14.5±0.4	17.0±0.4	16.8±0.4	12.2±0.2
28		50	16.4±0.3	20.2±0.6	17.6±0.4	12.2±0.3
29		55	20.7±0.6	20.6±0.4	18.0±0.4	16.3±0.3
30		60	22.5±0.5	20.7±0.5	18.3±0.3	16.3±0.6

Table S10. Effect of curing temperature on liquid resistance chromaticity variation of self-repairing brass powder-water-based acrylic decorative coatings.

Sample	Content of brass powder (%)	Curing temperature (°C)	Liquid resistance ΔE^*			
			Citric acid	Ethanol	Dishwashing detergent	Coffee

10		30	5.01±0.11	4.60±0.14	8.33±0.11	9.88±0.31
11		35	4.24±0.06	3.24±0.09	8.10±0.25	9.86±0.24
12		40	3.90±0.12	2.67±0.04	8.00±0.21	9.83±0.35
13	3	45	3.50±0.06	2.36±0.04	7.86±0.23	9.80±0.31
14		50	2.93±0.08	2.35±0.08	7.32±0.31	7.89±0.22
15		55	2.11±0.07	2.11±0.06	7.30±0.24	6.71±0.17
16		60	2.11±0.06	2.11±0.06	7.30±0.21	4.16±0.11
17		30	6.86±0.15	5.08±0.12	9.32±0.32	11.17±0.48
18		35	6.86±0.16	5.08±0.24	8.97±0.25	10.67±0.39
19		40	5.01±0.14	4.37±0.14	8.55±0.26	10.50±0.39
20	6	45	4.22±0.08	3.63±0.11	8.10±0.23	10.25±0.29
21		50	4.22±0.10	3.17±0.15	7.93±0.19	9.64±0.30
22		55	3.63±0.06	2.58±0.06	7.71±0.13	7.08±0.18
23		60	3.63±0.08	2.58±0.07	7.63±0.18	5.50±0.10
24		30	7.13±0.21	5.37±0.11	9.56±0.34	12.10±0.23
25		35	7.75±0.14	5.14±0.16	9.23±0.23	11.66±0.30
26		40	5.10±0.11	4.48±0.14	9.19±0.27	11.07±0.23
27	9	45	4.90±0.09	4.20±0.07	8.30±0.15	10.30±0.55
28		50	4.64±0.10	4.02±0.08	8.23±0.26	9.94±0.21
29		55	4.47±0.09	3.77±0.09	8.01±0.16	7.28±0.21
30		60	3.76±0.12	3.67±0.09	7.74±0.21	5.70±0.14
